# Supplementary material for: Molecular basis for the folding of β-helical autotransporter passenger domains
Source: Nat Commun. 2018 Apr 11;9:1395. doi: 10.1038/s41467-018-03593-2 (PMC5895577; doi:10.1038/s41467-018-03593-2)
Supplement: Supplementary file 1 — Supplementary Information(PDF 4611 kb) [file 41467_2018_3593_MOESM1_ESM.pdf]

**Molecular basis for the folding of β-helical autotransporter passenger domains**

Xiaojun Yuan[1#], Matthew D. Johnson[1#], Jing Zhang[1], Alvin W. Lo[2,3], Mark A. Schembri[2,3], Lakshmi C. Wijeyewickrema[4], Robert N. Pike[4], Gerard H. M. Huysmans[5], Ian R. Henderson[6], and Denisse L. Leyton[1,7]*

1 - Research School of Biology, Australian National University, Canberra, ACT, 0200, Australia

2 - School of Chemistry and Molecular Biosciences, The University of Queensland, Brisbane, Queensland, 4072, Australia

3 - Australian Infectious Disease Research Centre, The University of Queensland, Brisbane, Queensland, 4072, Australia

4 - Department of Biochemistry and Genetics, La Trobe Institute for Molecular Science, La Trobe University, Melbourne, 3086, VIC, Australia

5 - Department of Physiology and Biophysics, Weill Cornell Medicine, New York, NY 10065, U.S.A.

6 - Institute of Microbiology and Infection, University of Birmingham, Birmingham, B15 2TT, UK

7 - Medical School, Australian National University, Canberra, ACT, 0200, Australia

#These authors contributed equally to this work

*To whom correspondence should be addressed: denisse.leyton@anu.edu.au

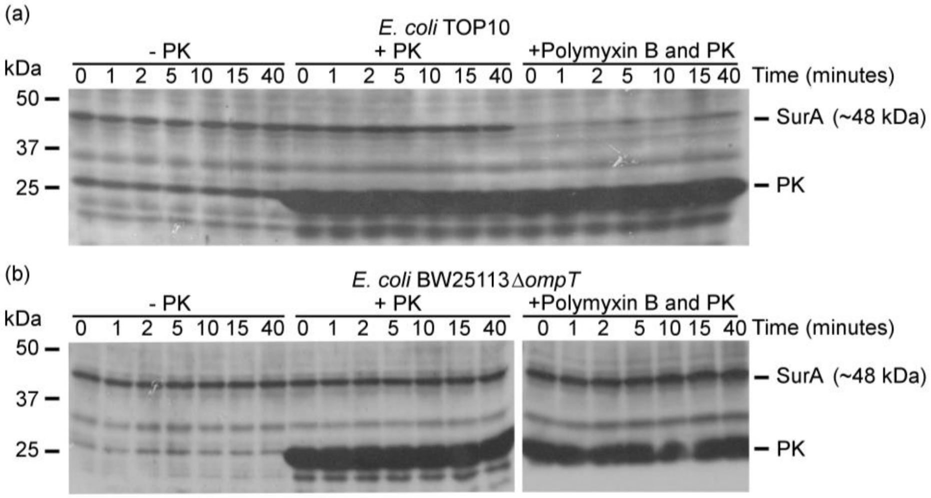


**Supplementary Figure 1 | Proteinase K does not permeabilize the outer membrane of *E. coli* TOP10 or *E. coli* BW25113Δ*ompT*. (a)** *E. coli* TOP10 and **(b)** *E. coli* BW25113Δ*ompT* incubated for up to 40 min of a mock 'chase' period at 25 °C and then immediately precipitated with TCA (- PK), or incubated on ice with 200 mg/mL proteinase K (+ PK), or incubated on ice with 200 mg/mL polymyxin B and 200 mg/mL proteinase K (+ Polymyxin B and PK). After addition of Phenylmethanesulfonyl fluoride (see Methods), proteinase K-treated samples were TCA precipitated and all samples were then analyzed by SDS-PAGE and immunoblotting for the periplasmic protein SurA. In both bacterial strains, the intact SurA protein (48 kDa) was observed in – PK and + PK samples. Note that a thick band corresponding to the migration position of proteinase K is also observed. These data show that the amount of proteinase K in use does not permeabilize the *E. coli* outer membrane, which remains intact during proteinase K treatment. Furthermore, SurA was degraded almost to completion by proteinase K in the presence of polymyxin B in *E. coli* TOP10. In contrast, intact SurA was observed in samples treated with proteinase K in the presence of polymyxin B in *E. coli* BW25113Δ*ompT*, indicating that 200 mg/mL polymyxin B is insufficient to permeabilize this strain's outer membrane. As a consequence, only *E. coli* TOP10 was used to perform pulse-chase assays in the presence of polymyxin B and proteinase K in this study. Images are representative of at least two independent experiments.

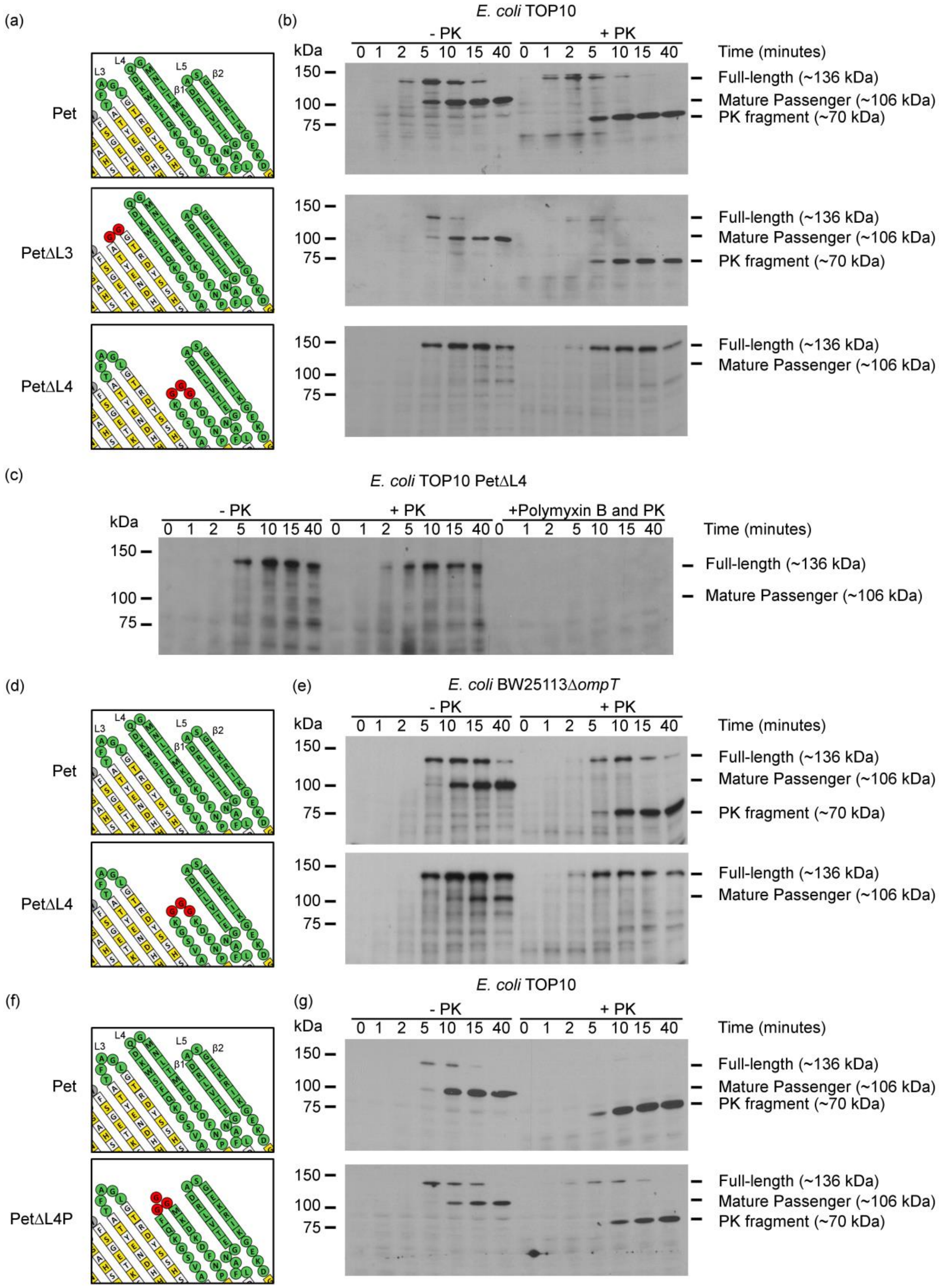
(a)
(b)
E. coli TOP10
- PK
+ PK
kDa
0 1 2 5 10 15 40 0 1 2 5 10 15 40
Time (minutes)
150
100
75
Full-length (~136 kDa)
Mature Passenger (~106 kDa)
PK fragment (~70 kDa)
L3
L4
L5
β1
β2
Pet
PetΔL3
PetΔL4
(c)
E. coli TOP10 PetΔL4
- PK
+ PK
+Polymyxin B and PK
0 1 2 5 10 15 40 0 1 2 5 10 15 40 0 1 2 5 10 15 40
(d)
(e)
E. coli BW25113ΔompT
(f)
(g)
E. coli TOP10
PetΔL4P

**Supplementary Figure 2 | Truncation of L4 perturbs passenger domain translocation. (a)** Topology model of the Pet β-barrel domain showing the L3 and L4 truncations created by replacing $T_{1130}$-$L_{1134}$ and $Q_{1177}$-$D_{1191}$ with two and with three glycine residues (shown in red), respectively. **(b)** Pulse-chase expression of Pet, PetΔL3 and PetΔL4, and sensitivity to proteinase K (PK) in *E. coli* TOP10. **(c)** Pulse-chase PetΔL4 maturation as above, but also in the presence of polymyxin B prior to the addition of proteinase K as indicated (+ Polymyxin B and PK). **(d)** Topology model of the Pet β-barrel domain showing the L4 truncation created by replacing $Q_{1177}$-$D_{1191}$ with three glycine residues (shown in red). **(e)** Pulse-chase expression of Pet and PetΔL4, and sensitivity to proteinase K (PK) in *E. coli* BW25113Δ*ompT*. **(f)** Topology model of the Pet β-barrel domain showing the partial L4 truncation created by replacing $S_{1179}$-$T_{1188}$ with three glycine residues (shown in red). **(g)** Pulse-chase expression of Pet and PetΔL4P, and sensitivity to proteinase K (PK) in *E. coli* TOP10. All samples were TCA precipitated prior to SDS-PAGE and immunoblotting with anti-Pet passenger domain antibodies. Images are representative of at least two independent experiments.

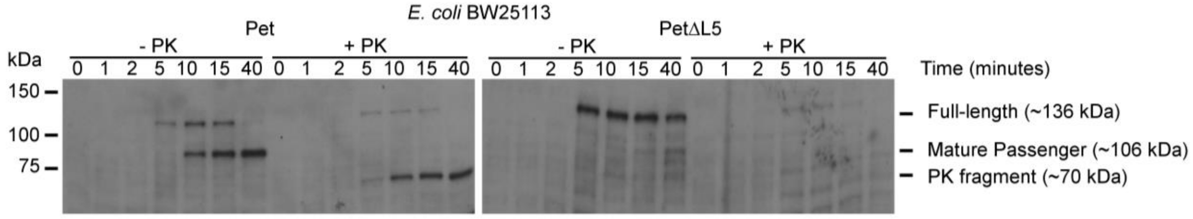


**Supplementary Figure 3 | Pet biogenesis in *E. coli* BW25113.** Pulse-chase expression of Pet and PetΔL5, and sensitivity to proteinase K (PK) in *E. coli* BW25113 monitored by SDS-PAGE and immunoblotting with anti-Pet passenger domain antibodies. All samples were TCA precipitated prior to SDS-PAGE. Image is representative of at least two independent experiments.

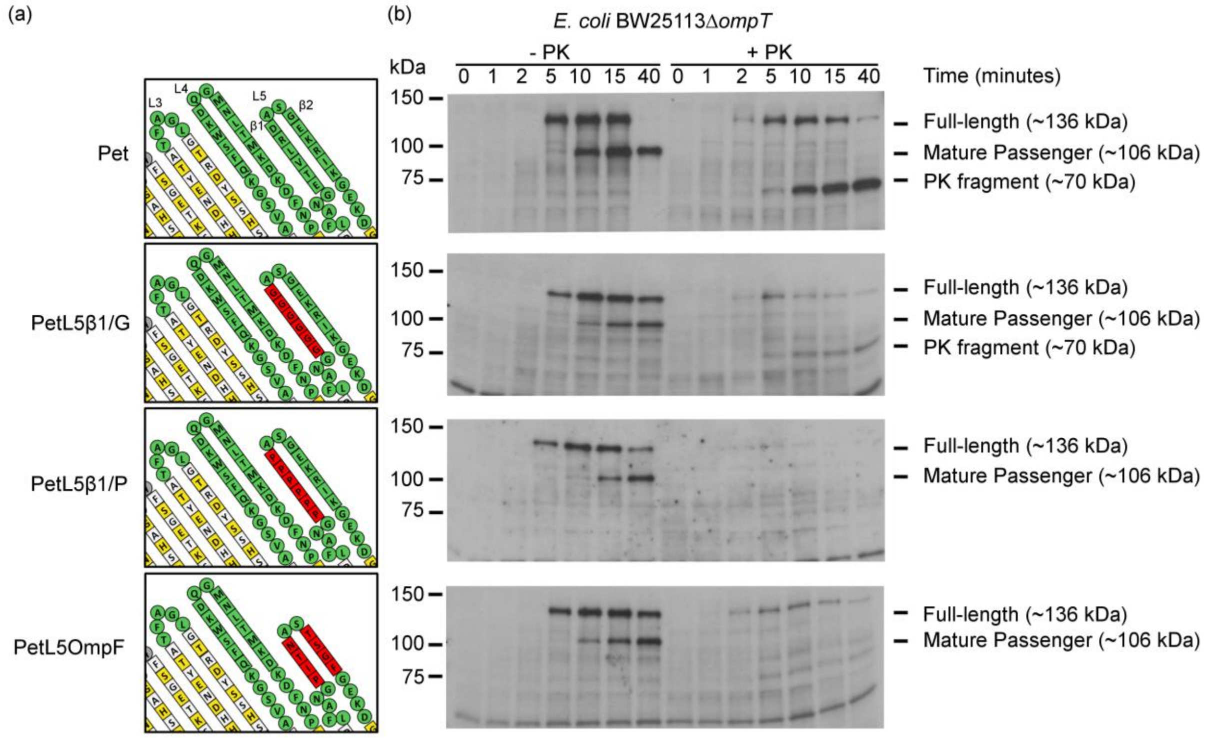


**Supplementary Figure 4 | Mutation of L5 affects folding of the Pet passenger domain. (a)** Topology model of the Pet β-barrel domain showing the $Pet^{L5\beta1/G}$, $Pet^{L5\beta1/P}$ and $Pet^{L5OmpF}$ mutations (in red). **(b)** Pulse-chase expression of Pet, $Pet^{L5\beta1/G}$, $Pet^{L5\beta1/P}$ and $Pet^{L5OmpF}$, and sensitivity to proteinase K (PK) in *E. coli* BW25113Δ*ompT* monitored by SDS-PAGE and immunoblotting with anti-Pet passenger domain antibodies. All samples were TCA precipitated prior to SDS-PAGE. Images are representative of at least two independent experiments.

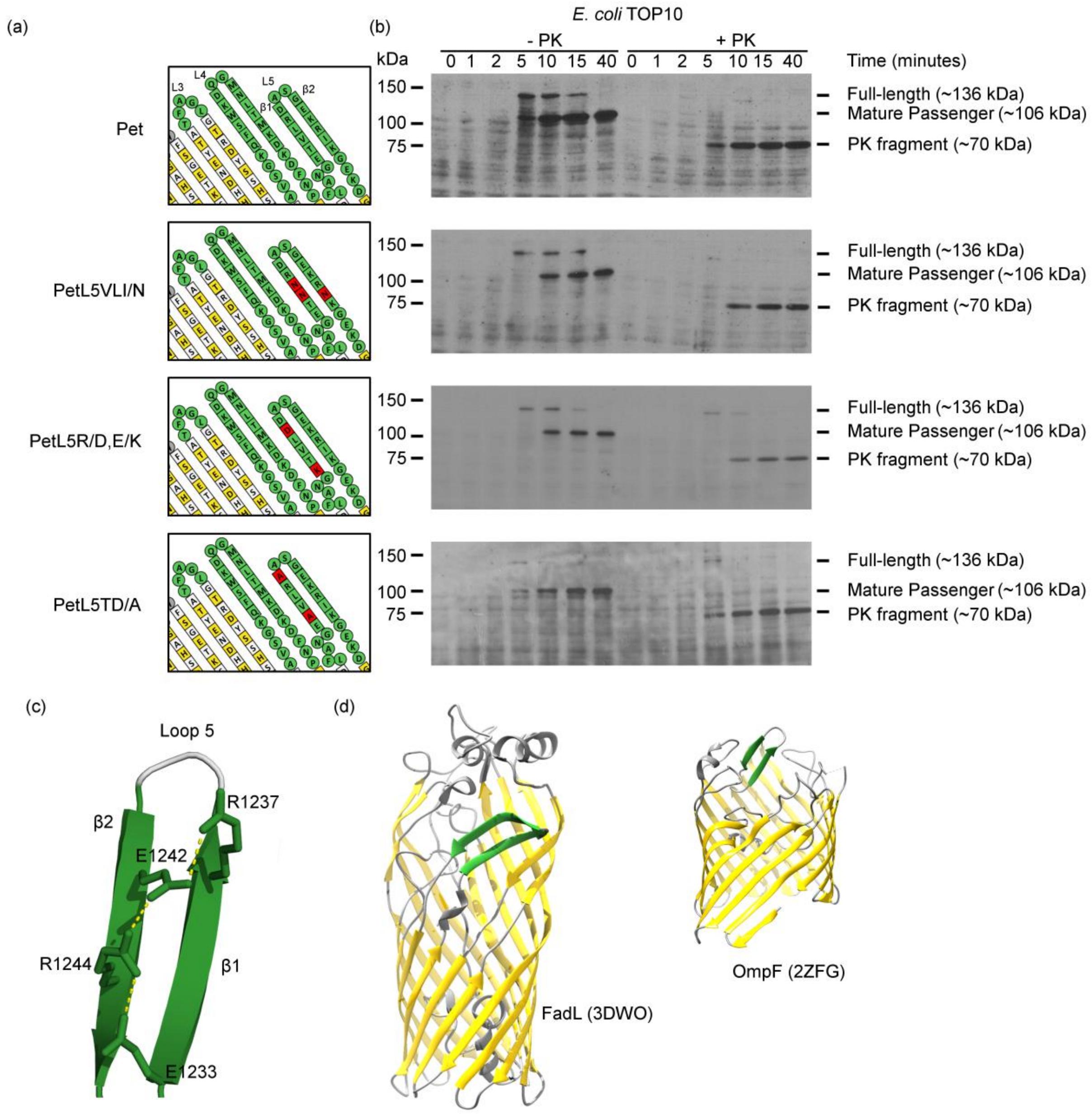


**Supplementary Figure 5 | Hydrophobic and charged residues do not mediate passenger folding. (a)** Topology model of the Pet β-barrel domain showing the $Pet^{L5VLI/N}$, $Pet^{L5R/D,E/K}$ and $Pet^{L5TD/A}$ mutations (in red). **(b)** Pulse-chase expression of Pet, $Pet^{L5VLI/N}$, $Pet^{L5R/D,E/K}$ and $Pet^{L5TD/A}$, and sensitivity to proteinase K (PK) in *E. coli* TOP10 monitored by SDS-PAGE and immunoblotting with anti-Pet passenger domain antibodies. All samples were TCA precipitated prior to SDS-PAGE. Images are representative of at least two independent experiments. **(c)** The crystal structure of the EspP β-barrel domain (PDB code 3SLJ) showing putative salt-bridge interactions between $R_{1237}$ in β-strand 1 and $E_{1242}$ in β-strand 2 (top of L5), and a $E_{1233}$ in β-strand 1 and $R_{1244}$ in β-strand 2 (bottom of L5). **(d)** The crystal structures of FadL from *Pseudomonas aeruginosa* (PDB code 3DWO) and OmpF from *E. coli* (PDB code 2ZFG). In each case, the β-strands within the β-hairpin loop are shown in green.

(a)

```
Pet         LGY------QFDLFA-NGETVLRDASGEKRIKGEK----DGRMLMNVGLNAEIRDN-VRF
EspP        LGY------QFDLLA-NGETVLRDASGEKRIKGEK----DSRMLMSVGLNAEIRDN-VRF
Tsh/Hbp     LHY------EFDLTD-SADVHLKDAAGEHQINGRK----DSRMLYGVGLNARFGDN-TRL
Vat         LHY------EFDLTD-SADVHLKDAAGEHQINGRK----DGRMLYGVGLNARFGDN-TRL
Boa         VDY------QFDLVA-NGETALRDASGEKRFTGEK----DSRMLYNVGLNAQVKDN-VRF
Pic         TSW------QFDLLN-NGETVLRDASGEKRIKGEK----DSRMLFNVGMNAQIKDN-MRF
SigA        LGY------QFDLFA-NGETVLRDASGEKRIKGEK----DGRILMNVGLNAEIRDN-LRF
EspI        LGY------QFDLLA-NGETVLRDASGEKRIKGEK----DSRMLMSVGLNAEIRDN-VRF
EpeA        LGY------QFDLLA-NGETVLRDASGEKRIKGEK----DSRMLMSVGLNAEIRDN-VRF
EaaA        LGY------QFDLLA-NGETVLRDASGEKRIKGEK----DGRMLMSVGLNAEVRDN-IRF
Sat         LGY------QFDLFA-NGETVLRDASGEKRIKGEK----DGRMLMNVGLNAEIRDN-LRF
EatA        LGY------QFDLLA-NGETVLQDASGKKHFKGEK----DSRMLMNVGTNVEVKDN-MRF
EspC        LGY------QFDLLA-NGETVLRDASGEKRFEGEK----DSRMLMNVGMNAEIKDN-MRF
SepA        LGY------QFDLLA-NGETVLQDASGEKRFEGEK----DSRMLMTVGMNAEIKDN-MRL
TibA        AAV------SHEFSD-NNKVRINDTYDFRNDISGT----TGK--YGLGVNAQLTPN-AGV
BapA        VNV------KHEFLD-GTRVR---VAGVPVSSRMA----RTWGSVGVGADYGWGER-YAI
VacA        VLQ------EFANFG-SSNAVSLNTFKVNAVRNPL----NTHARVMMGGELKLAKE-VFL
EstA        HER------EYEDDT-QDLTMSLNSLPGNRFTLEGYTPQDHLNRVSLGFSQKLAPE-LSL
TapA        TGY------AGTLKVAQVETVGLTSTTETGLVTP-----NGALDTGAGVTLRGHHTPWTV
PspA        LGW------QHSLSAVESEEHLAFVAGGPSFAVQSSPLMRDAALVGVQASLALSKS-TRV
NalP        VERDLN---GRDYTVTGGFTGATAATGKTGARNMP----HTRLVAGLGADVEFGNG-WN-
Ag43        VNWWVQPSVIRTFSSRGDMRVGTSTAGSGMTFSPSQN--GTSLDLQAGLEARVREN-ITL
IcsA/VirG   VNW------KWSSKQ-YGVIM----NGMSNHQIGN----RNVIELKTGVGGRLADN-LSI
AIDA        ANW------IH-----NTHEFGVKMSDDSQLLSGS----RNQGEIKTGIEGVITQN-LSV
IgA1        AAY-------FANYGKGGVNV-----GGKSFAYKA----DNQQQYSAGVALLYRNVTLNV
BrkA        LGW------TQEFKS-TGDVR---TNGIGHAGAGR----HGRVELGAGVDAALGKG-HNL
```

(b)

| | | | Tsh/Hbp | Vat | Boa | Pic | SigA | EspI | EspP | EpeA | EaaA | Sat | Pet | EatA | EspC | SepA |
|---|---|---|---|---|---|---|---|---|---|---|---|---|---|---|---|---|
| | | | 1 | 2 | 3 | 4 | 5 | 6 | 7 | 8 | 9 | 10 | 11 | 12 | 13 | 14 |
| Tsh/Hbp | 1 | (Type_5) | 100.0 | 98.92 | 62.09 | 62.45 | 60.65 | 61.01 | 61.73 | 62.09 | 62.45 | 62.09 | 62.45 | 59.21 | 62.09 | 61.01 |
| Vat | 2 | (Type_5) | 98.92 | 100.0 | 61.37 | 62.45 | 61.37 | 60.65 | 61.37 | 61.73 | 62.82 | 62.45 | 62.82 | 59.21 | 62.09 | 61.01 |
| Boa | 3 | (Type_5) | 62.09 | 61.37 | 100.0 | 71.84 | 68.59 | 68.59 | 69.68 | 69.68 | 68.59 | 68.23 | 68.95 | 64.98 | 70.04 | 67.15 |
| Pic | 4 | (Type_5) | 62.45 | 62.45 | 71.84 | 100.0 | 79.06 | 80.14 | 80.14 | 80.14 | 80.87 | 79.06 | 79.78 | 71.48 | 79.42 | 77.26 |
| SigA | 5 | (Type_5) | 60.65 | 61.37 | 68.59 | 79.06 | 100.0 | 86.28 | 87.73 | 87.36 | 88.09 | 88.81 | 89.17 | 71.84 | 81.23 | 78.70 |
| EspI | 6 | (Type_5) | 61.01 | 60.65 | 68.59 | 80.14 | 86.28 | 100.0 | 96.75 | 96.39 | 90.97 | 88.81 | 89.89 | 72.56 | 79.42 | 77.26 |
| EspP | 7 | (Type_5) | 61.73 | 61.37 | 69.68 | 80.14 | 87.73 | 96.75 | 100.0 | 99.64 | 92.42 | 89.89 | 90.97 | 73.65 | 81.23 | 79.06 |
| EpeA | 8 | (Type_5) | 62.09 | 61.73 | 69.68 | 80.14 | 87.36 | 96.39 | 99.64 | 100.0 | 92.78 | 90.25 | 91.34 | 73.65 | 81.59 | 79.42 |
| EaaA | 9 | (Type_5) | 62.45 | 62.82 | 68.59 | 80.87 | 88.09 | 90.97 | 92.42 | 92.78 | 100.0 | 92.42 | 93.14 | 74.37 | 81.95 | 79.42 |
| Sat | 10 | (Type_5) | 62.09 | 62.45 | 68.23 | 79.06 | 88.81 | 88.81 | 89.89 | 90.25 | 92.42 | 100.0 | 98.92 | 72.92 | 80.14 | 77.98 |
| Pet | 11 | (Type_5) | 62.45 | 62.82 | 68.95 | 79.78 | 89.17 | 89.89 | 90.97 | 91.34 | 93.14 | 98.92 | 100.0 | 73.65 | 80.87 | 78.70 |
| EatA | 12 | (Type_5) | 59.21 | 59.21 | 64.98 | 71.48 | 71.84 | 72.56 | 73.65 | 73.65 | 74.37 | 72.92 | 73.65 | 100.0 | 83.39 | 80.87 |
| EspC | 13 | (Type_5) | 62.09 | 62.09 | 70.04 | 79.42 | 81.23 | 79.42 | 81.23 | 81.59 | 81.95 | 80.14 | 80.87 | 83.39 | 100.0 | 94.22 |
| SepA | 14 | (Type_5) | 61.01 | 61.01 | 67.15 | 77.26 | 78.70 | 77.26 | 79.06 | 79.42 | 79.42 | 77.98 | 78.70 | 80.87 | 94.22 | 100.0 |

(c)

| | | | EstA | VacA | NalP | Ag43 | TapA | PspA | IgA1 | IcsA/VirG | AIDA | BrkA | TibA/TynE | BapA |
|---|---|---|---|---|---|---|---|---|---|---|---|---|---|---|
| | | | 1 | 2 | 3 | 4 | 5 | 6 | 7 | 8 | 9 | 10 | 11 | 12 |
| EstA | 1 | (Type_4) | 100.0 | 15.30 | 16.01 | 12.78 | 14.03 | 18.73 | 12.88 | 16.30 | 15.99 | 14.49 | 13.62 | 14.03 |
| VacA | 2 | (Type_3) | 15.30 | 100.0 | 11.66 | 15.65 | 13.26 | 16.73 | 15.13 | 15.30 | 14.55 | 15.90 | 15.79 | 11.64 |
| NalP | 3 | (Type_6) | 16.01 | 11.66 | 100.0 | 13.74 | 15.27 | 17.73 | 16.04 | 13.11 | 13.86 | 16.30 | 15.30 | 13.98 |
| Ag43 | 4 | (Type_13) | 12.78 | 15.65 | 13.74 | 100.0 | 14.06 | 15.47 | 14.46 | 17.10 | 15.30 | 20.15 | 19.62 | 19.62 |
| TapA | 5 | (Type_2) | 14.03 | 13.26 | 15.27 | 14.06 | 100.0 | 19.00 | 15.06 | 12.36 | 14.29 | 16.48 | 13.55 | 12.88 |
| PspA | 6 | (Type_1) | 18.73 | 16.73 | 17.73 | 15.47 | 19.00 | 100.0 | 16.79 | 19.03 | 16.04 | 20.30 | 16.79 | 17.52 |
| IgA1 | 7 | (Type_8) | 12.88 | 15.13 | 16.04 | 14.46 | 15.06 | 16.79 | 100.0 | 17.18 | 15.65 | 17.75 | 24.01 | 15.93 |
| IcsA/VirG | 8 | (Type_12) | 16.30 | 15.30 | 13.11 | 17.10 | 12.36 | 19.03 | 17.18 | 100.0 | 37.46 | 18.08 | 20.37 | 18.51 |
| AIDA | 9 | (Type_13) | 15.99 | 14.55 | 13.86 | 15.30 | 14.29 | 16.04 | 15.65 | 37.46 | 100.0 | 17.34 | 20.74 | 17.08 |
| BrkA | 10 | (Type_10) | 14.49 | 15.90 | 16.30 | 20.15 | 16.48 | 20.30 | 17.75 | 18.08 | 17.34 | 100.0 | 26.37 | 21.00 |
| TibA/TynE | 11 | (Type_10) | 13.62 | 15.79 | 15.30 | 19.62 | 13.55 | 16.79 | 24.01 | 20.37 | 20.74 | 26.37 | 100.0 | 21.75 |
| BapA | 12 | (Type_14) | 14.03 | 11.64 | 13.98 | 19.62 | 12.88 | 17.52 | 15.93 | 18.51 | 17.08 | 21.00 | 21.75 | 100.0 |

**Supplementary Figure 6 | Multiple sequence alignment and percent identity of autotransporter β-barrel domains. (a)** Multiple sequence alignment (performed using Muscle[1]) on the amino acid sequence of β-barrel domains that are representative of 11 distinct Types. The residues corresponding to L5 of EspP (PDB 3SLJ), Tsh/Hbp (PDB 3AEH), EstA (PDB 3KVN), AIDA-I (PDB 4MEE), NalP (PDB 1UYN), and BrkA (PDB 3QQ2) are shown in blue font and mapped according to their crystal structures. The L5 regions in SPATE β-barrels (shown in black font and underlined) are the same length and contain several residues that are highly or completely conserved (highlighted in yellow), particularly within the regions that form a β-hairpin (shown in red font). The L5 regions in non-SPATE β-barrel domains (shown in black font) differ substantially with each other and with those in SPATEs. **(b)** Percent identity between SPATE β-barrel domains ranges from 59.21- to- 99.64 %. **(c)** Percent identity between non-SPATE β-barrel domains ranges from 11.64- to- 37.46 % and even those belonging to the same Type share low sequence identity.

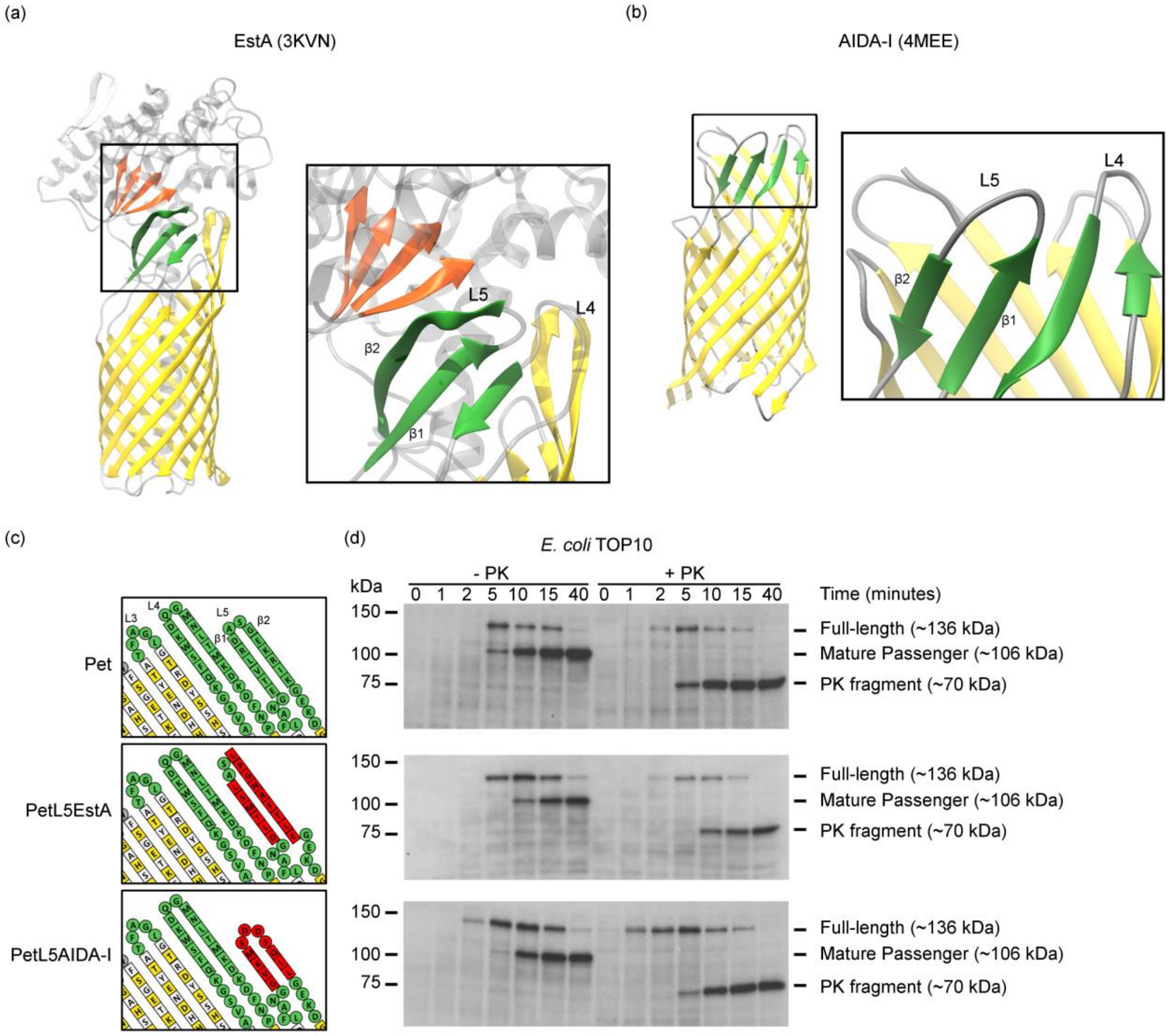


**Supplementary Figure 7 | Structural analysis of the AIDA-I and EstA β-barrel domains.** The crystal structures of EstA from *P. aeruginosa* (PDB code 3KVN) **(a)** and AIDA-I from *E. coli* (PDB code 4MEE) **(b)**. In each case, the β-strands within the L5 and L4 β-hairpins are shown in green. The central four-stranded parallel β-sheet within the globular passenger domain of EstA is shown in orange. **(c)** Topology model of the Pet β-barrel domain showing the $Pet^{L5EstA}$ and $Pet^{L5AIDA\text{-}I}$ mutations (in red). **(d)** Pulse-chase expression of $Pet^{L5EstA}$ and $Pet^{L5AIDA\text{-}I}$, and sensitivity to proteinase K (PK) in *E. coli* TOP10 monitored by SDS-PAGE and immunoblotting with anti-Pet passenger domain antibodies. All samples were TCA precipitated prior to SDS-PAGE. Images are representative of at least two independent experiments.

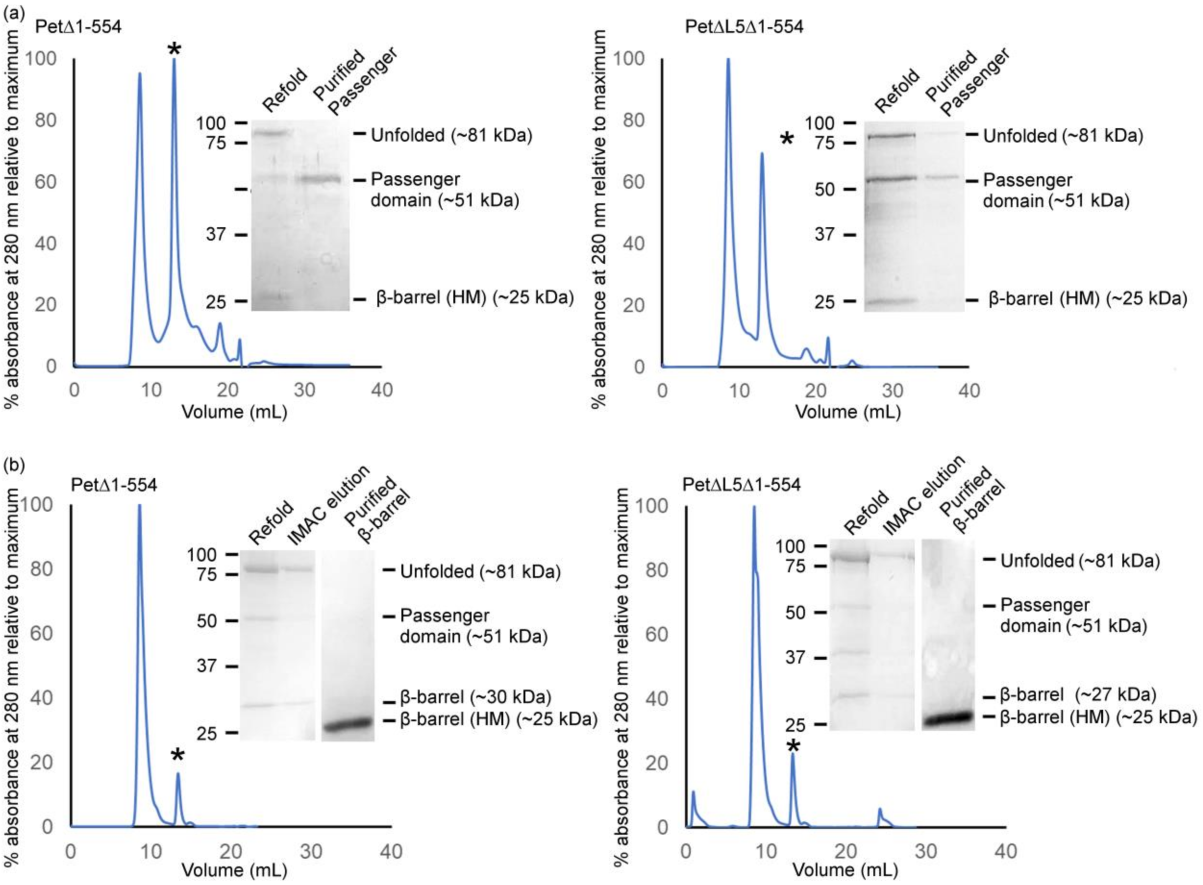


**Supplementary Figure 8 | Purification of Pet$^{\Delta 1\text{-}554}$ and PetΔL5$^{\Delta 1\text{-}554}$. (a)** Cleaved passenger domains were purified further by gel filtration in LDAO free buffer (* indicates the passenger domain elution peak), resulting in a batch of protein containing just the ~51 kDa species as analysed by SDS-PAGE and Coomassie staining (insets). **(b)** Cleaved β-barrel domains were purified further by gel filtration chromatography in buffer containing 0.05% (w/v) LDAO (* indicates the β-barrel elution peak), resulting in a batch of protein containing just the cleaved ~30 kDa species as analysed by SDS-PAGE and Coomassie staining (inset). Heat modifiability (HM) in the *in vitro*-folded Pet$^{\Delta 1\text{-}554}$ and PetΔL5$^{\Delta 1\text{-}554}$ β-barrels was evident by the increased migration of the folded species from samples exposed to SDS at 25 °C.

**Figure 1e Pet**

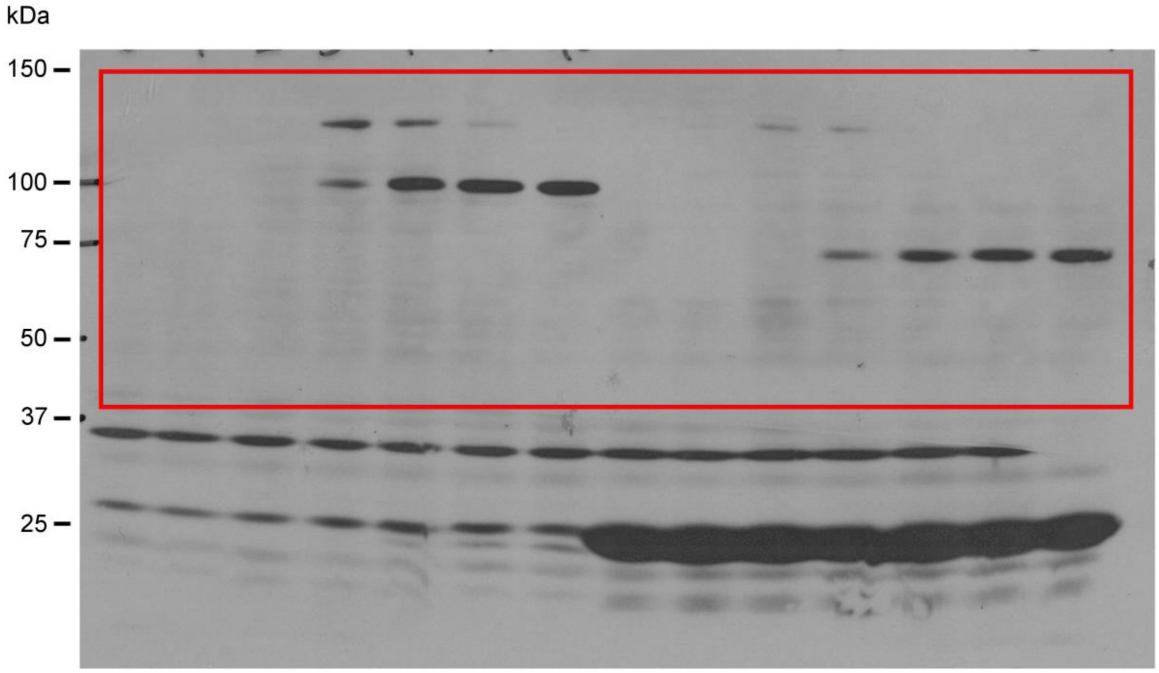


**Figure 1e PetΔL5**

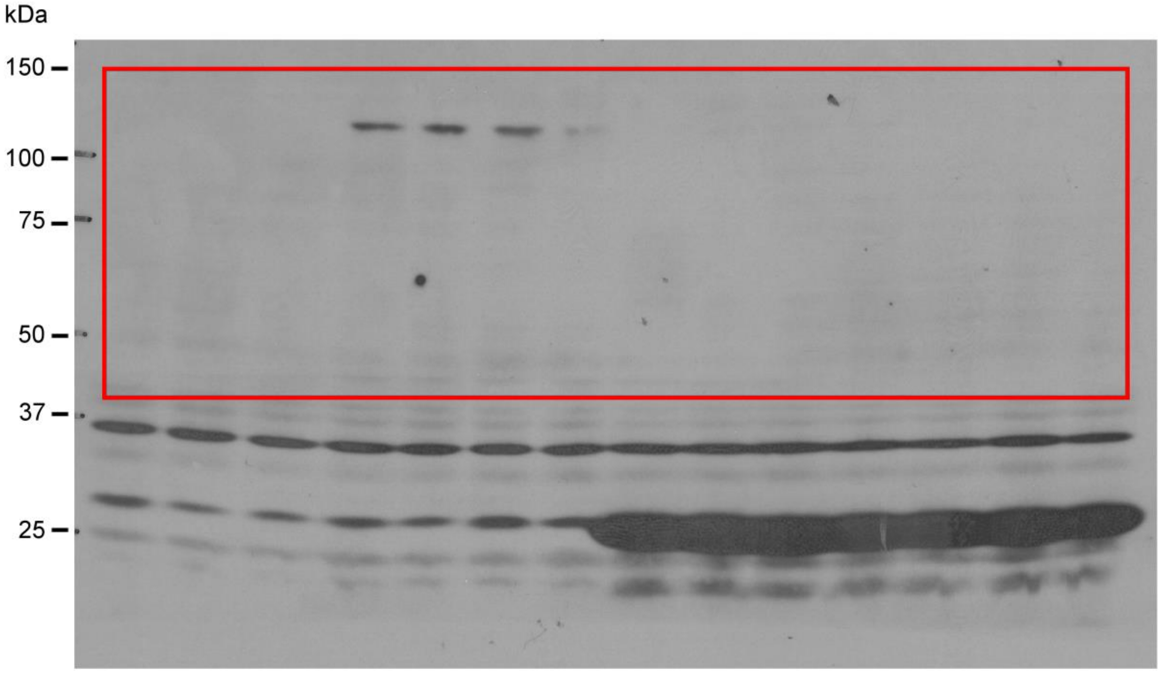

**Figure 1f Pet**

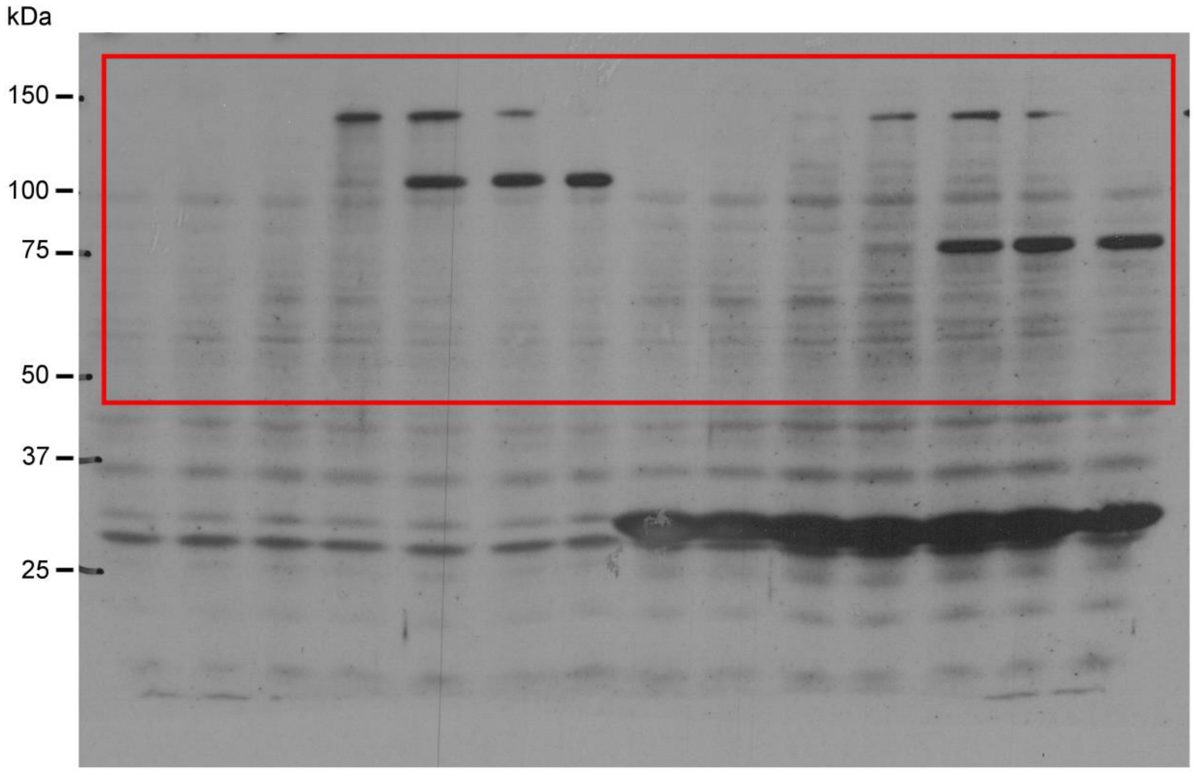


**Figure 1f PetΔL5**

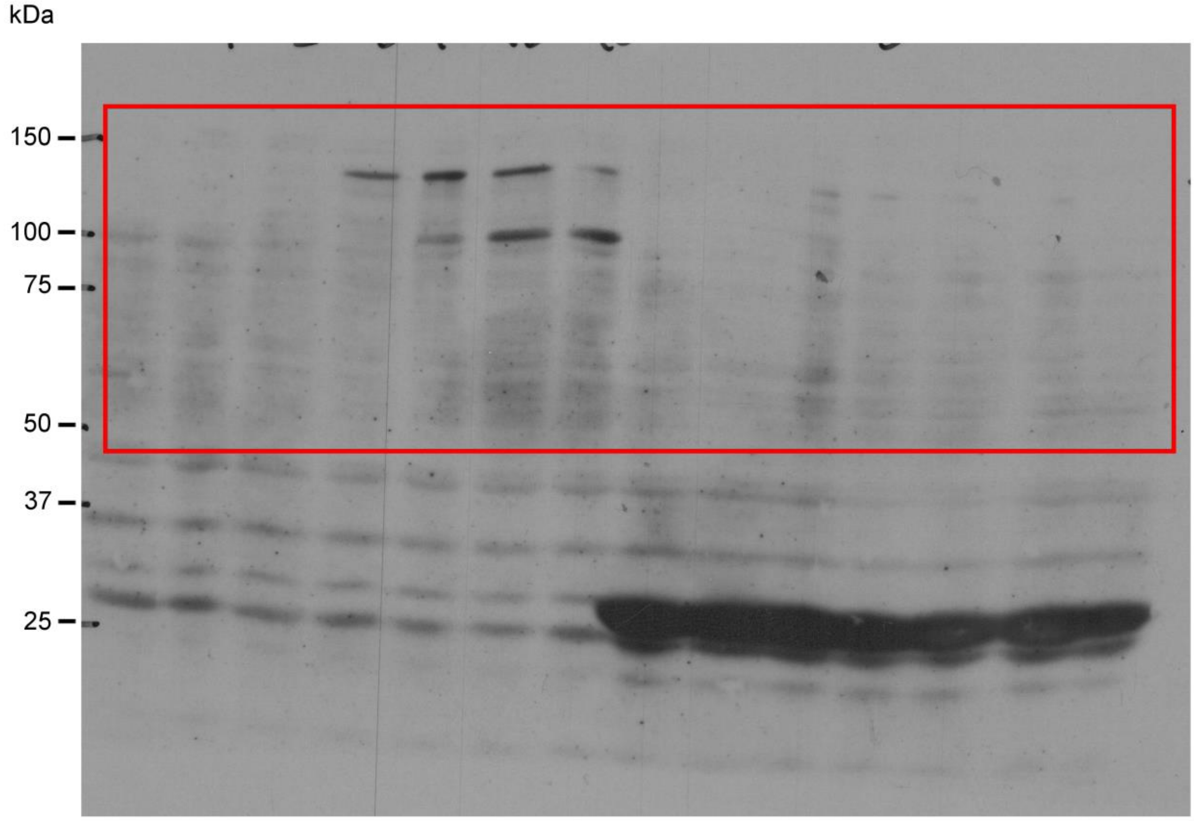

**Figure 1g**

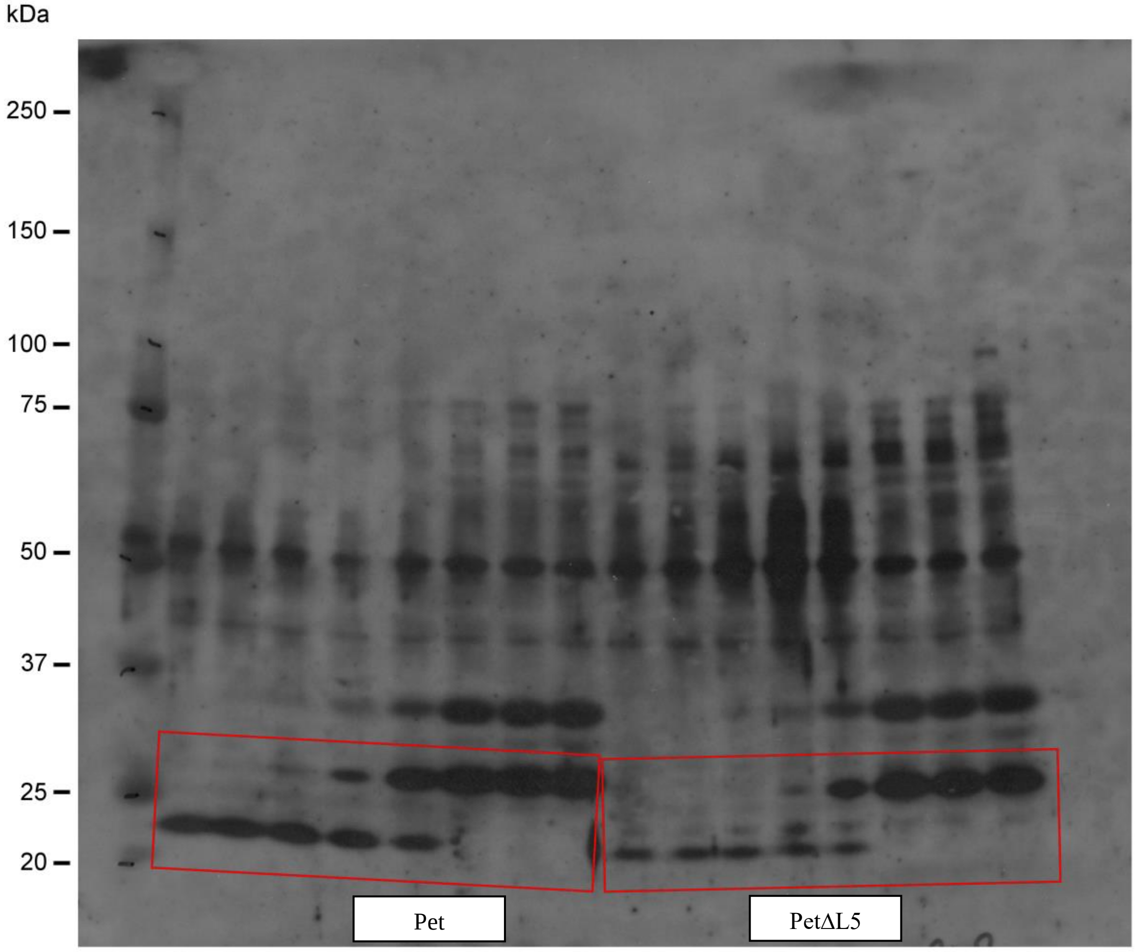


**Figure 2c Pet**

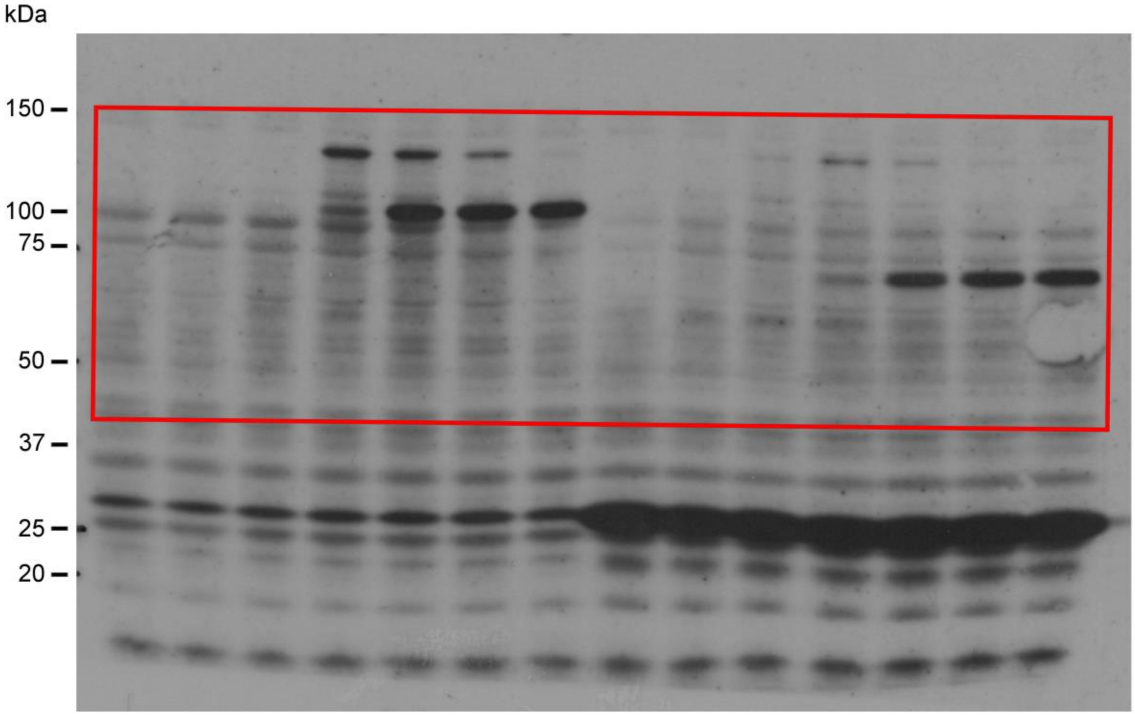

**Figure 2c PetL5β1/G**

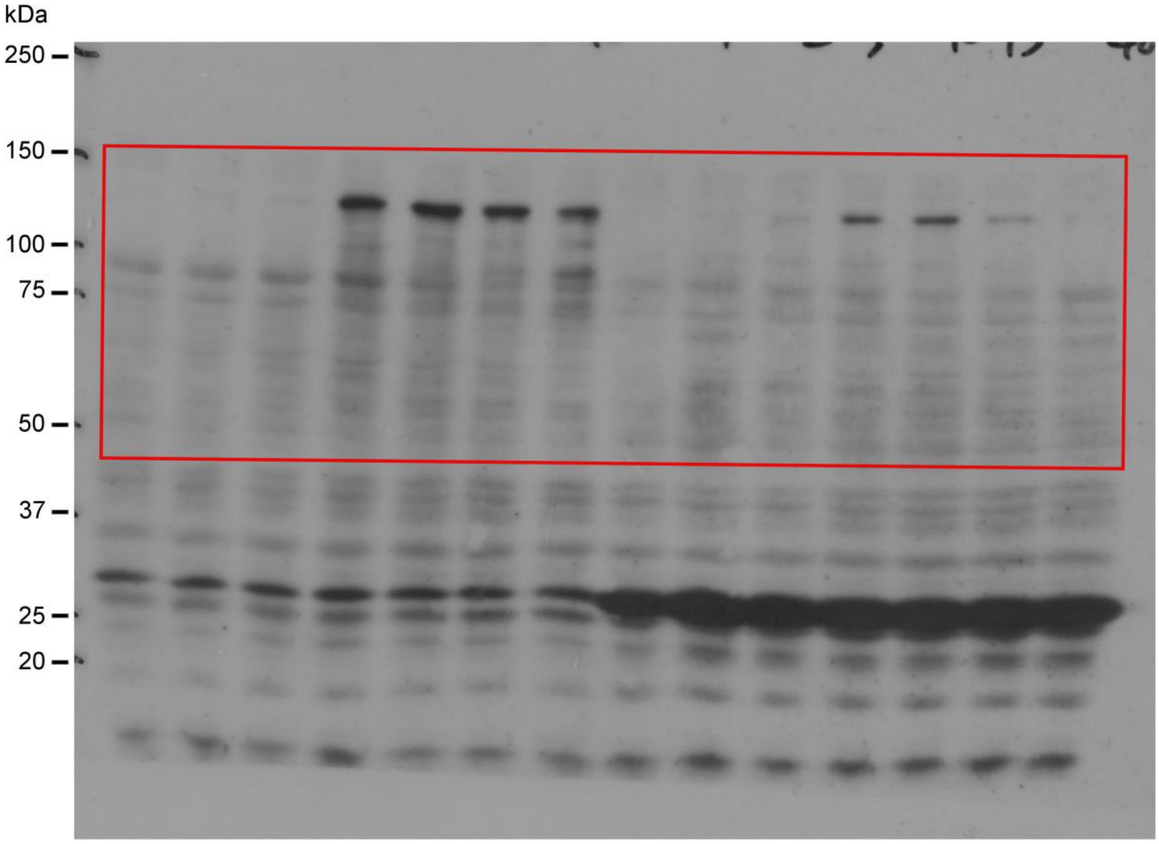


**Figure 2c PetL5β2/G**

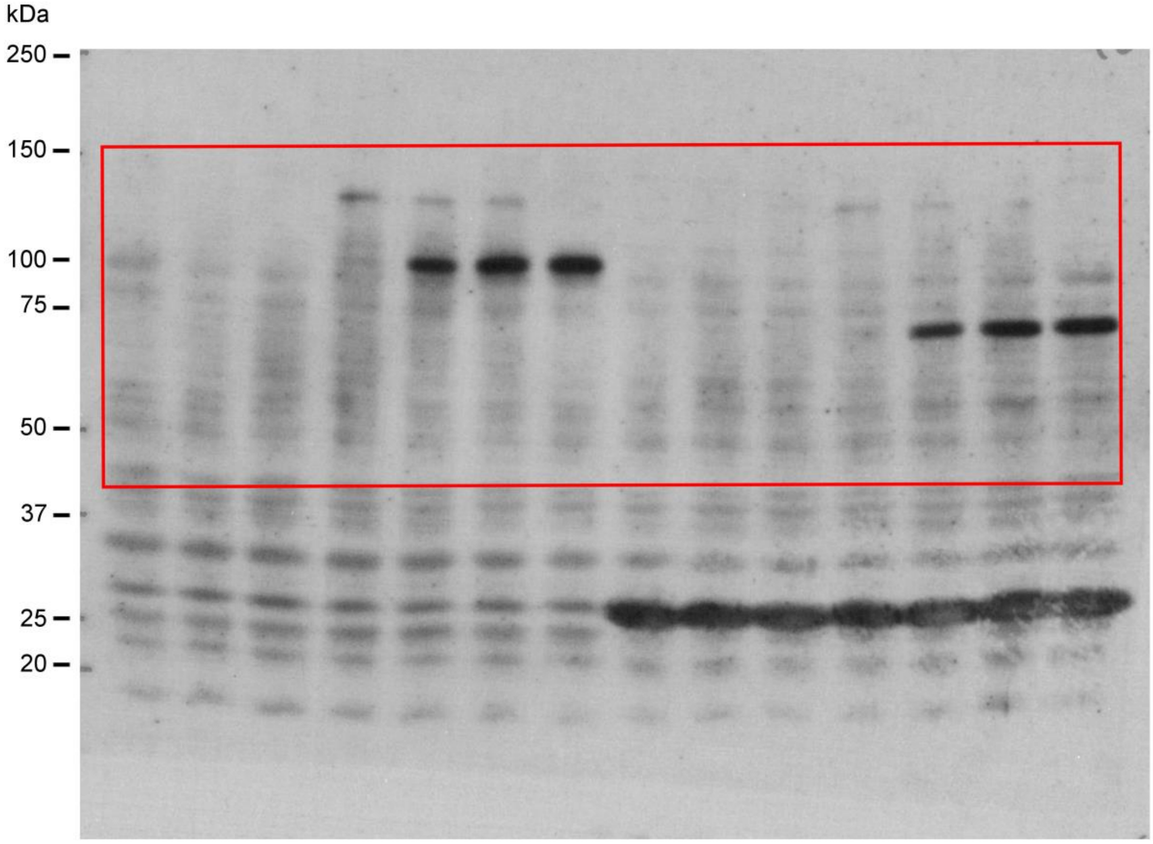

**Figure 2c PetL5Un/G**

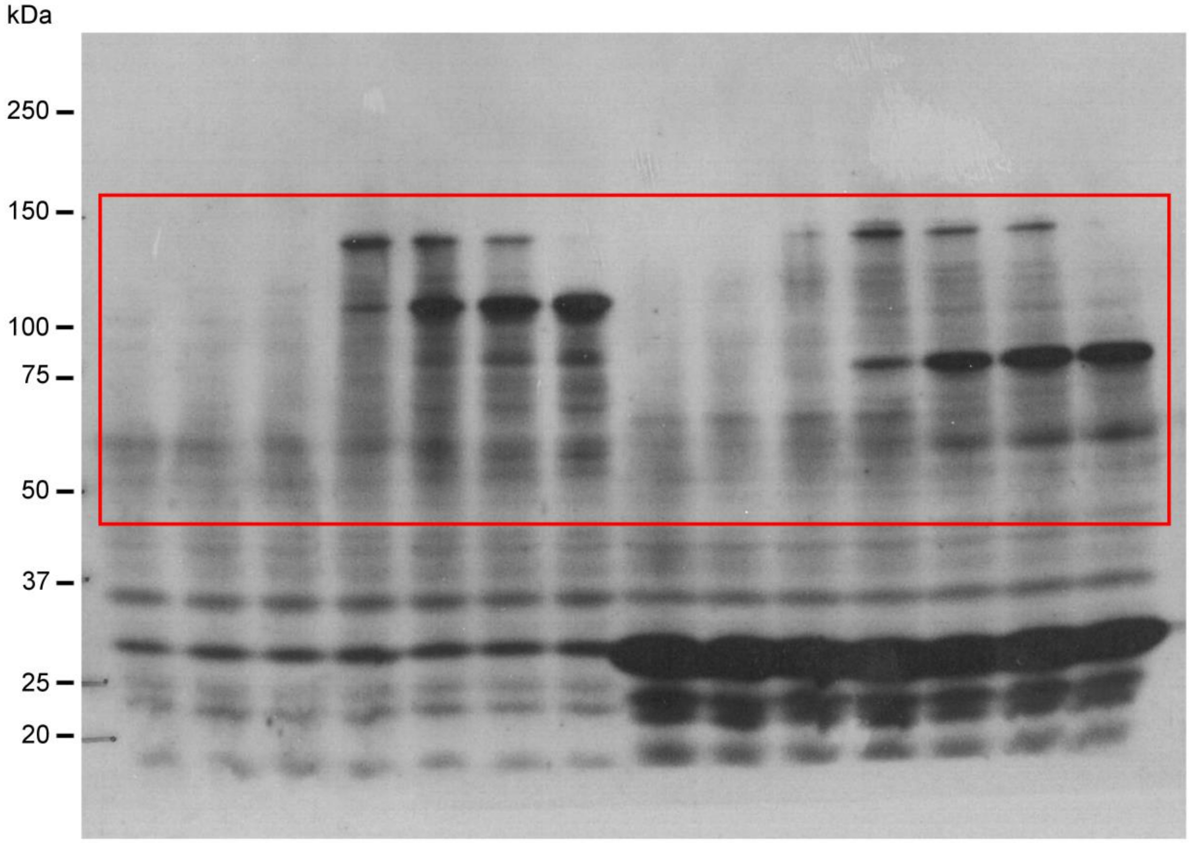


**Figure 2d Pet**

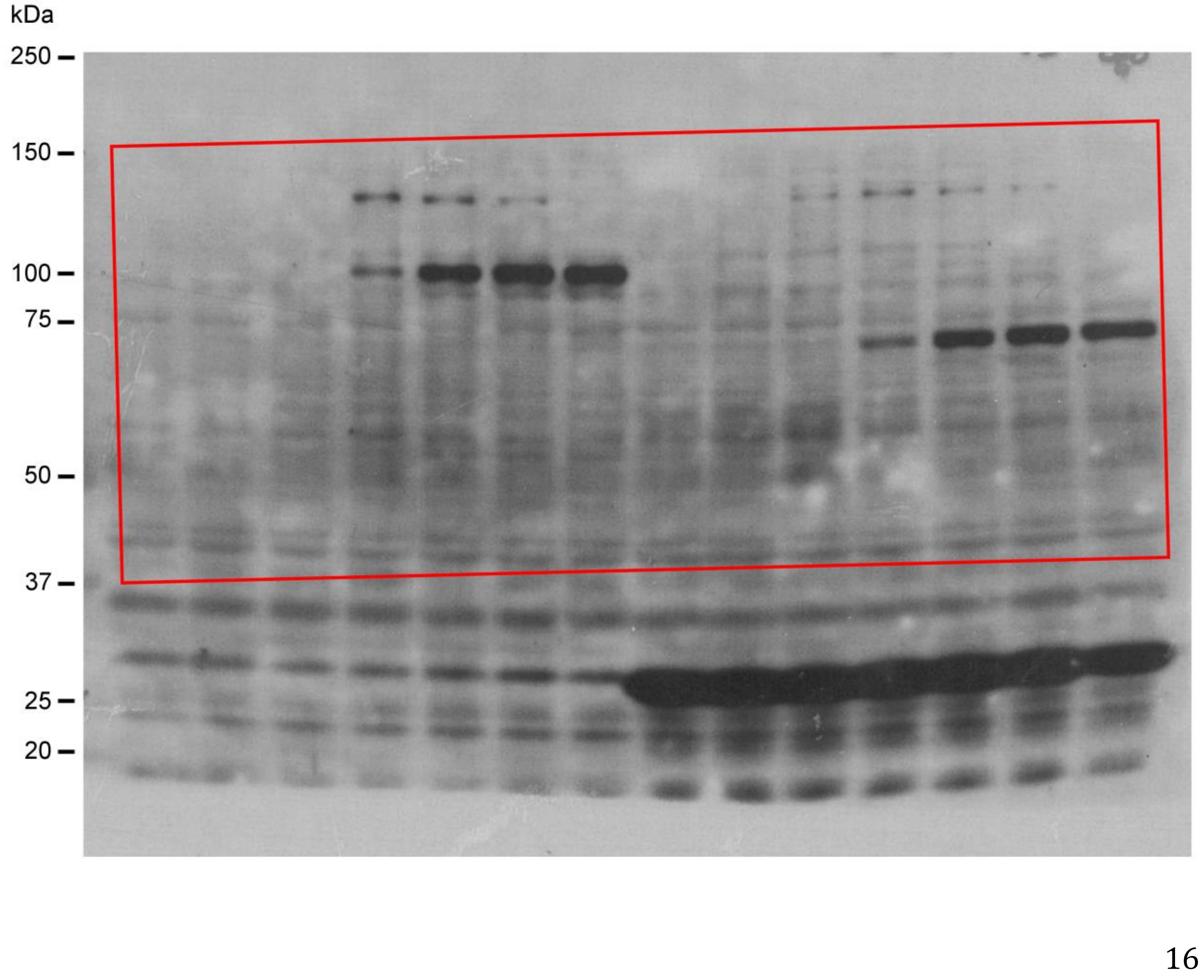

**Figure 2d PetL5OmpF**

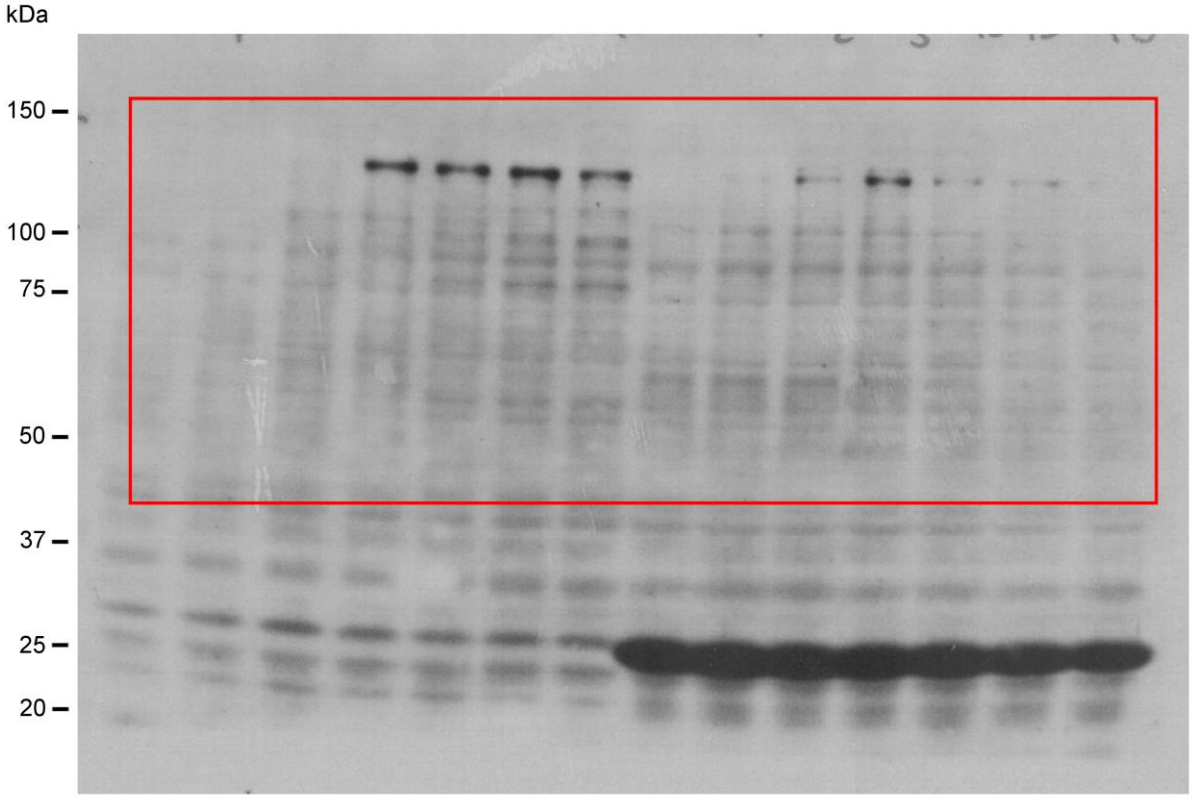


**Figure 2d PetL5FadL**

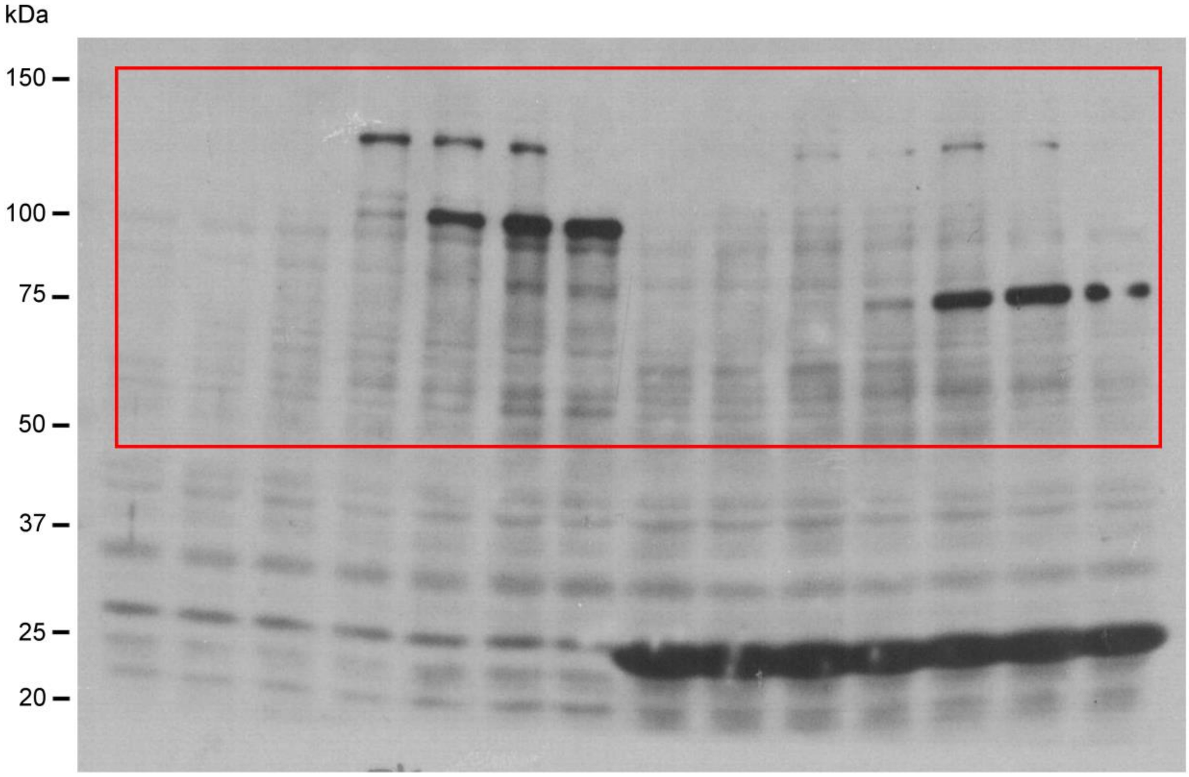

**Figure 2d PetL5β1/P**

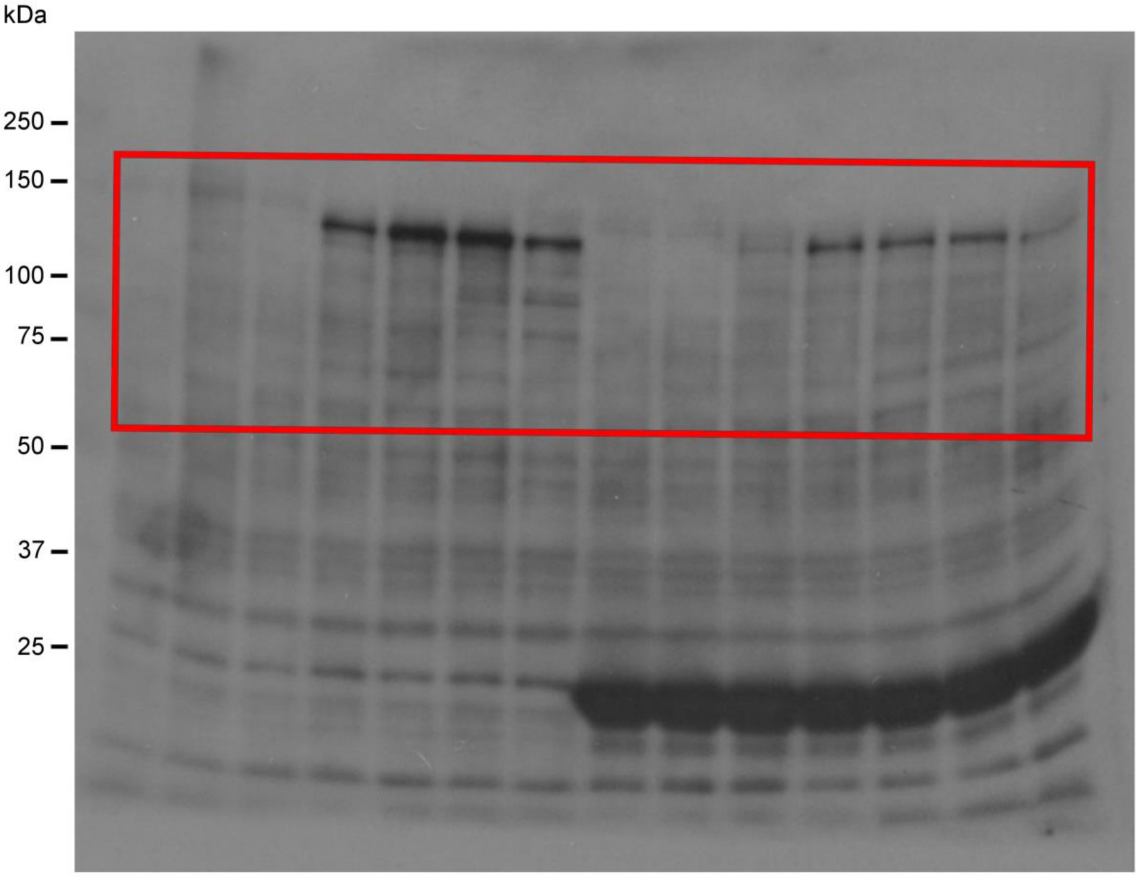


**Figure 3b top**

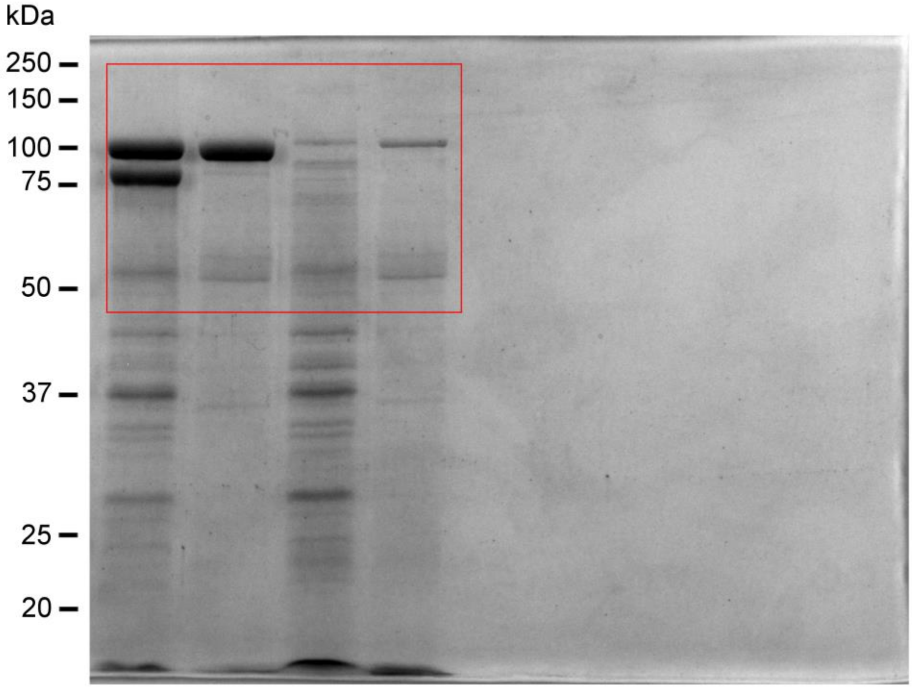

**Figure 3b bottom**

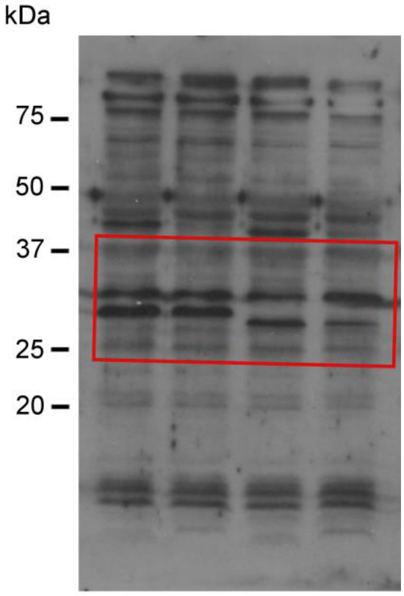


**Figure 4b**

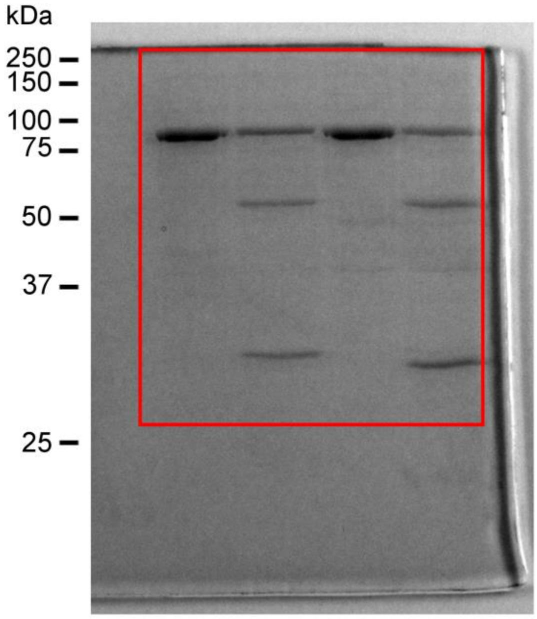

**Figure 4c Passenger antibody (top)**

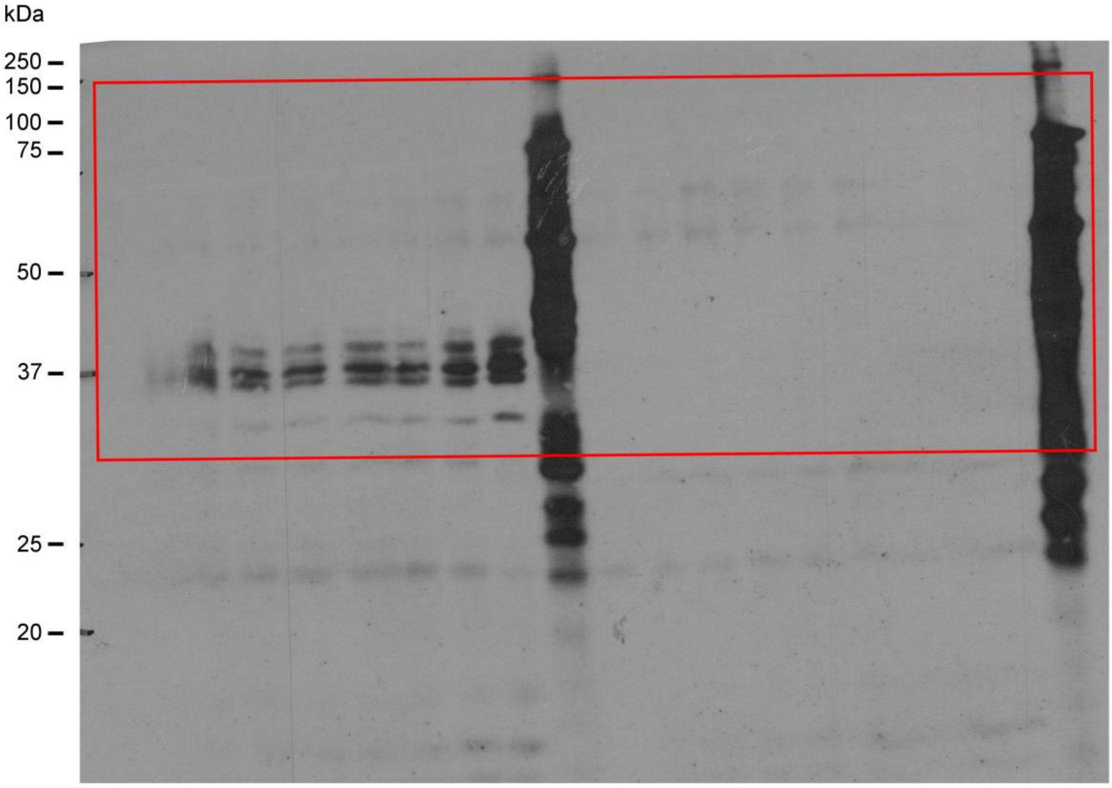


**Figure 4c β-barrel antibody (bottom)**

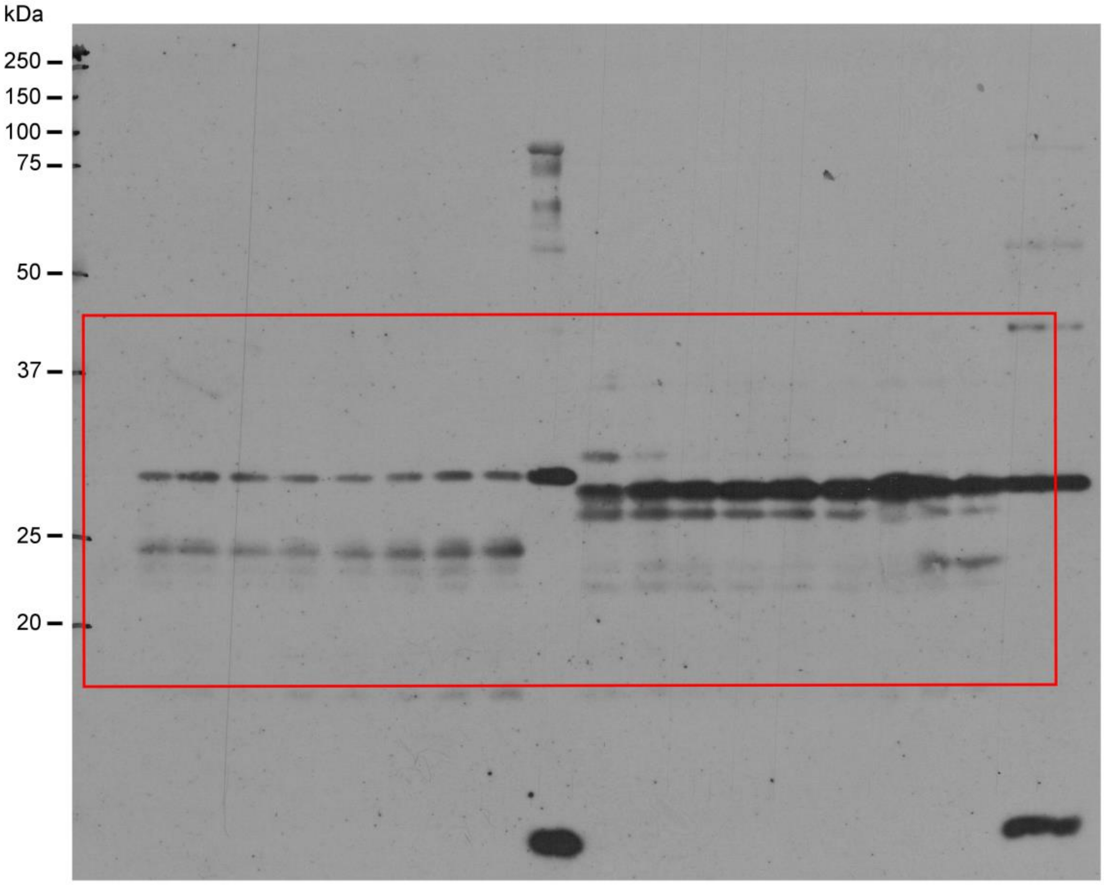

**Supplementary Figure 1a**

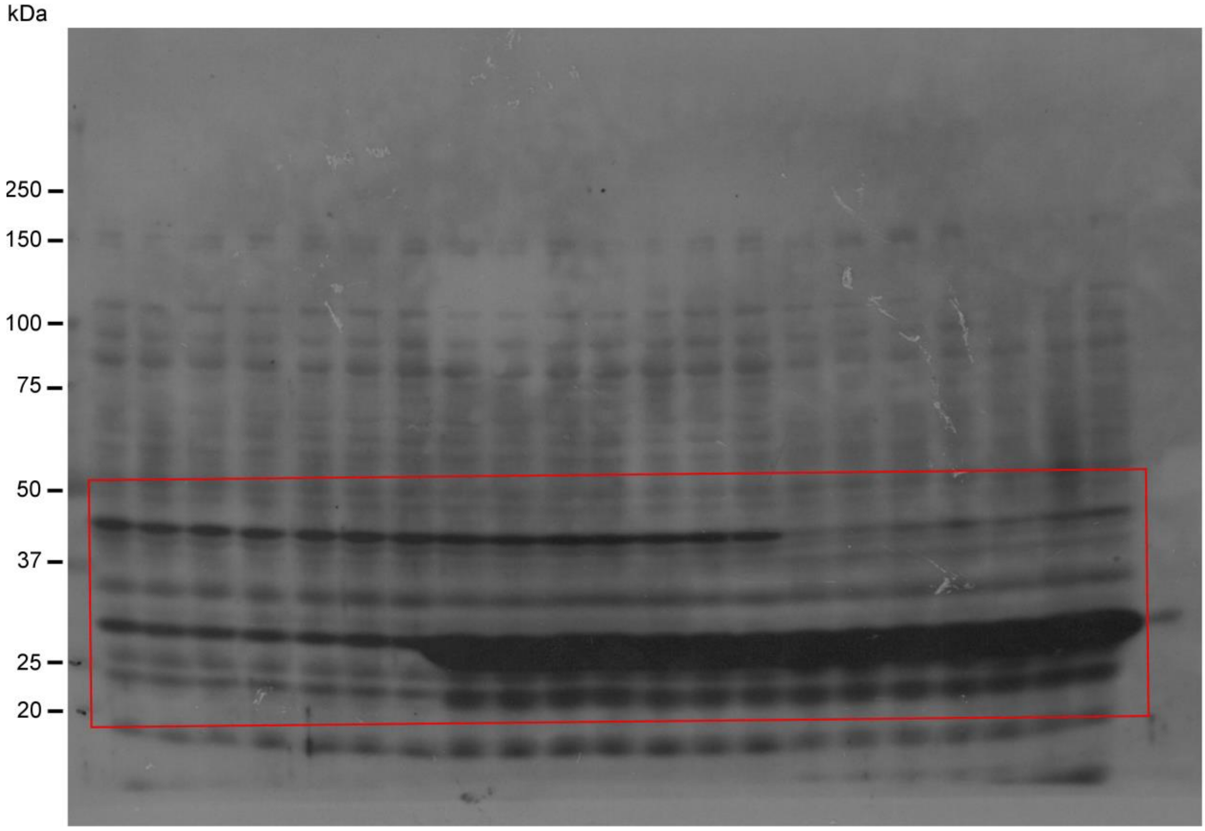


**Supplementary Figure 1b (left panel)**

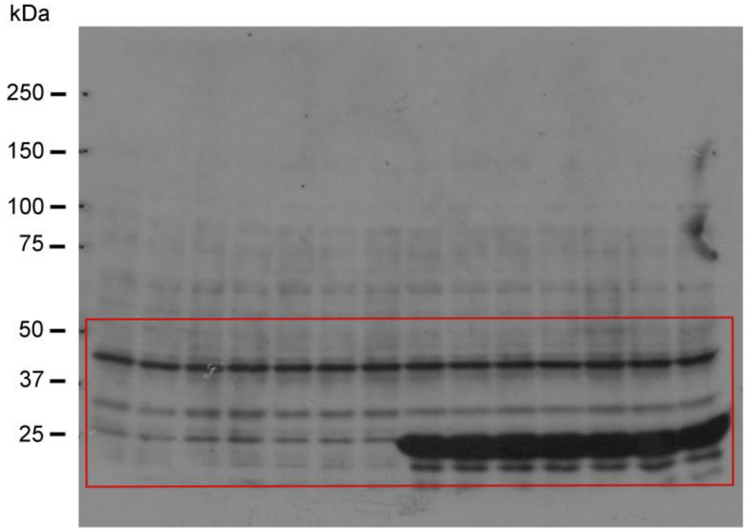


**Supplementary Figure 1b (right panel)**

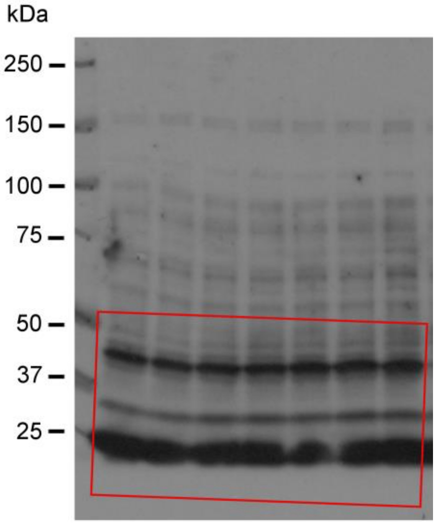

**Supplementary Figure 2b Pet**

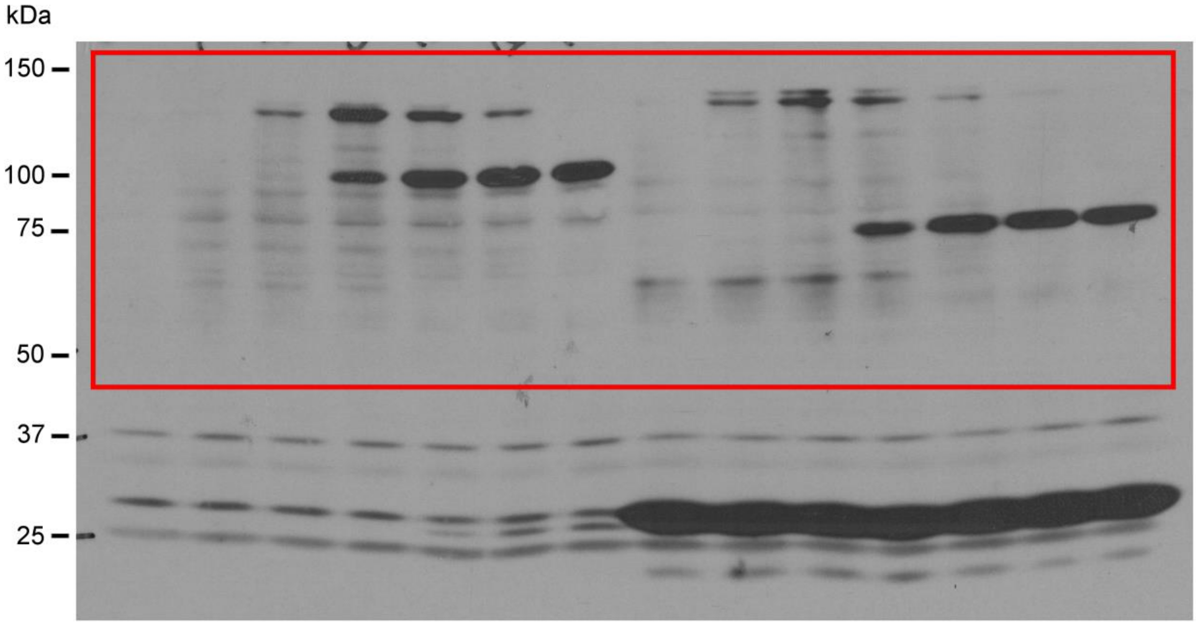


**Supplementary Figure 2b PetΔL3**

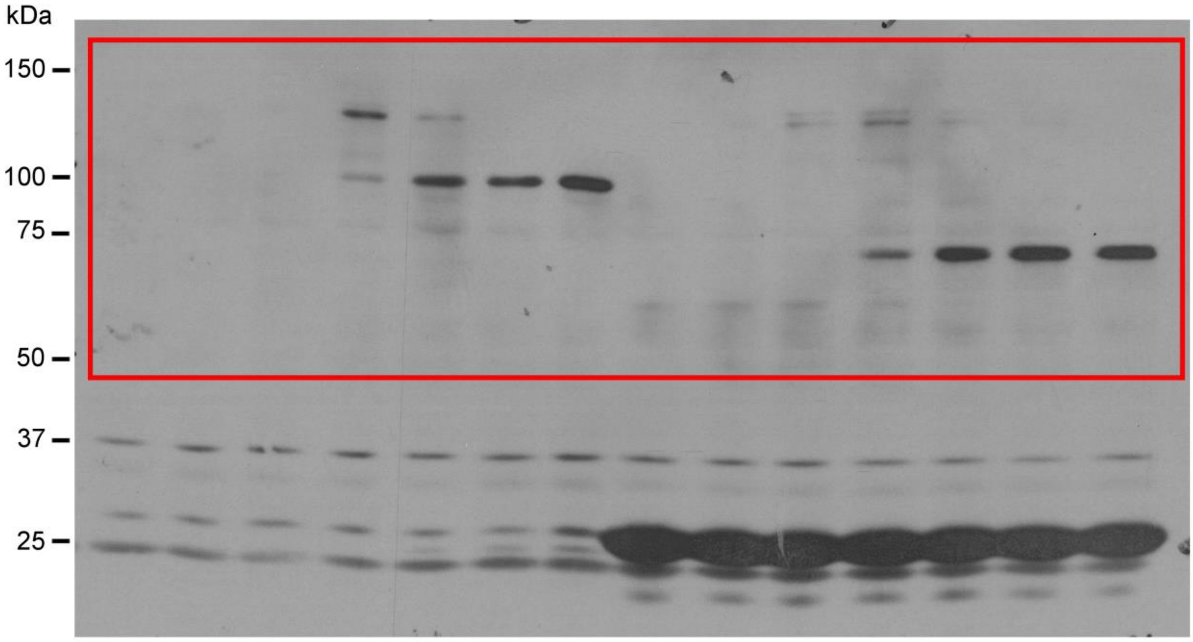

**Supplementary Figure 2b PetΔL4**

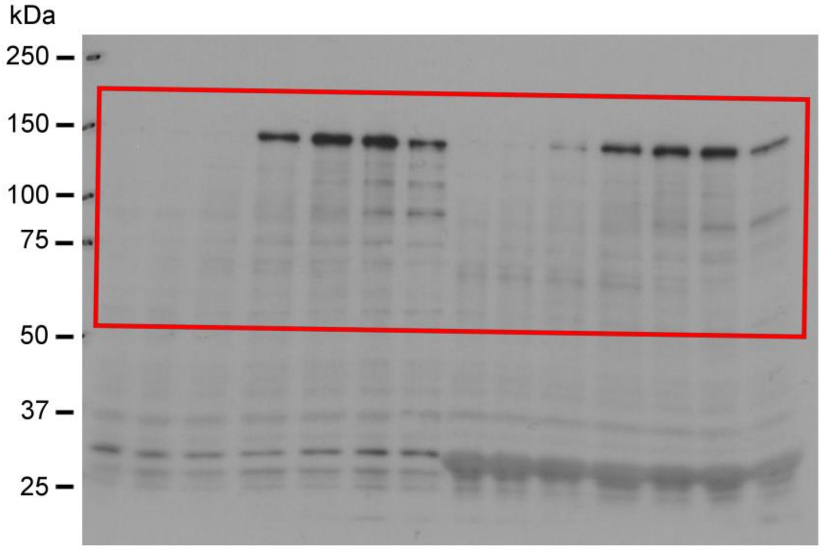


**Supplementary Figure 2c PetΔL4**

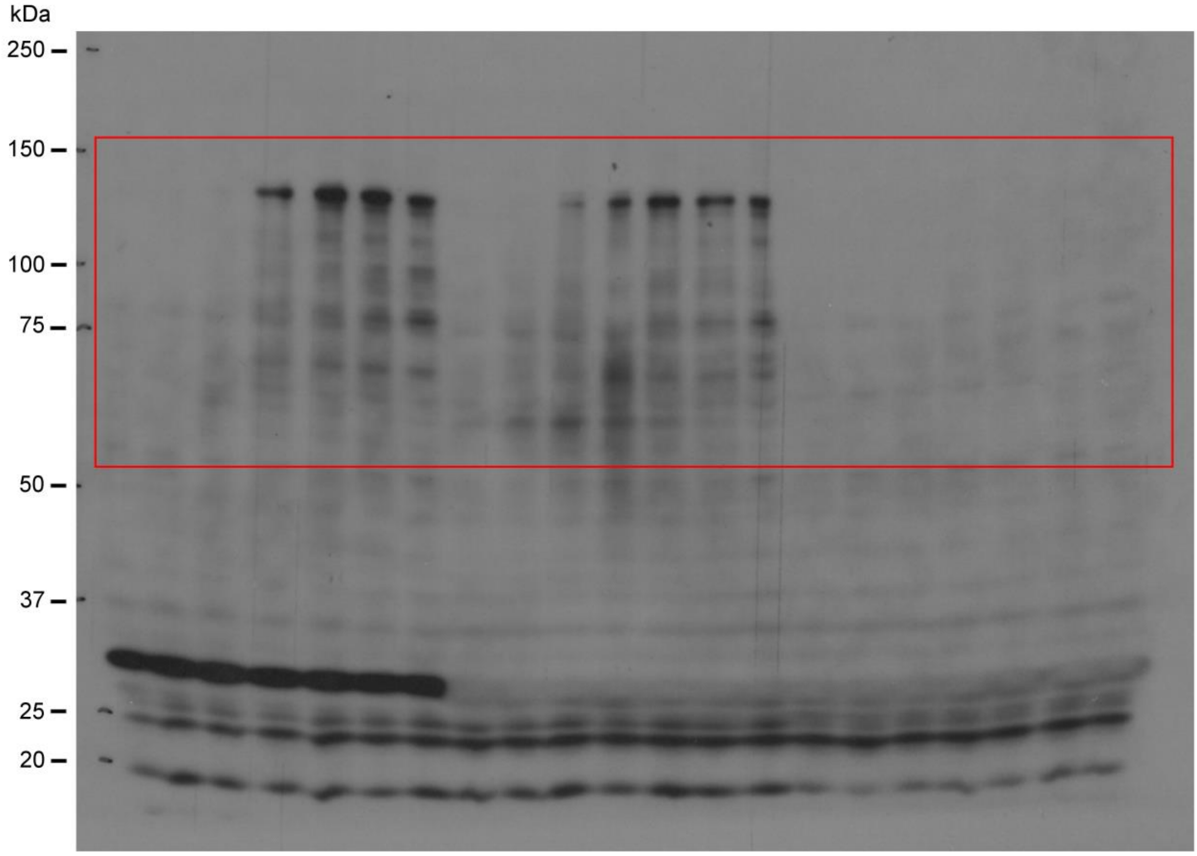

**Supplementary Figure 2e Pet**

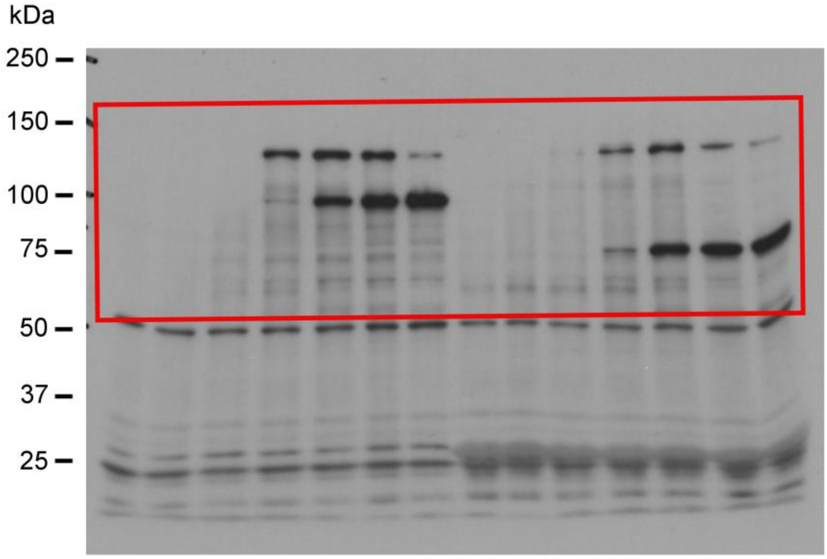


**Supplementary Figure 2e PetΔL4**

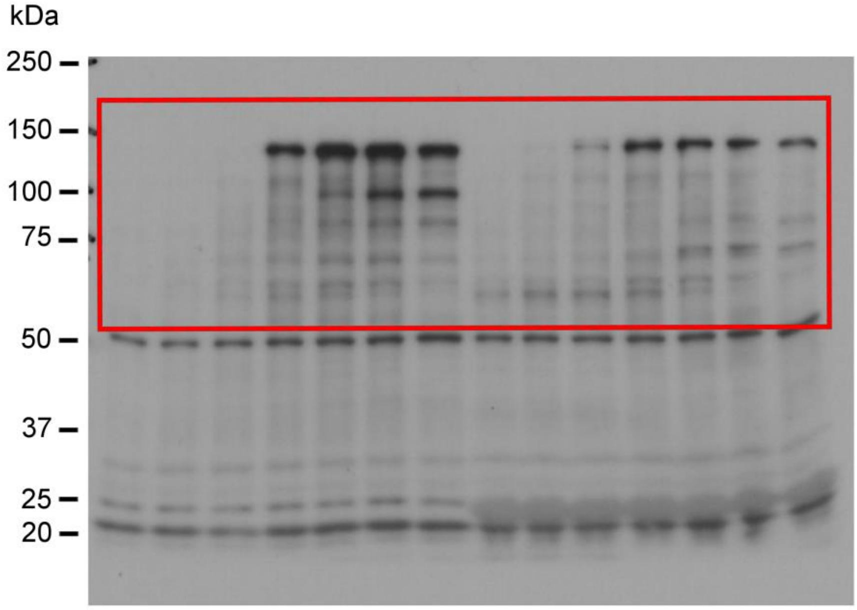

**Supplementary Figure 2g Pet**

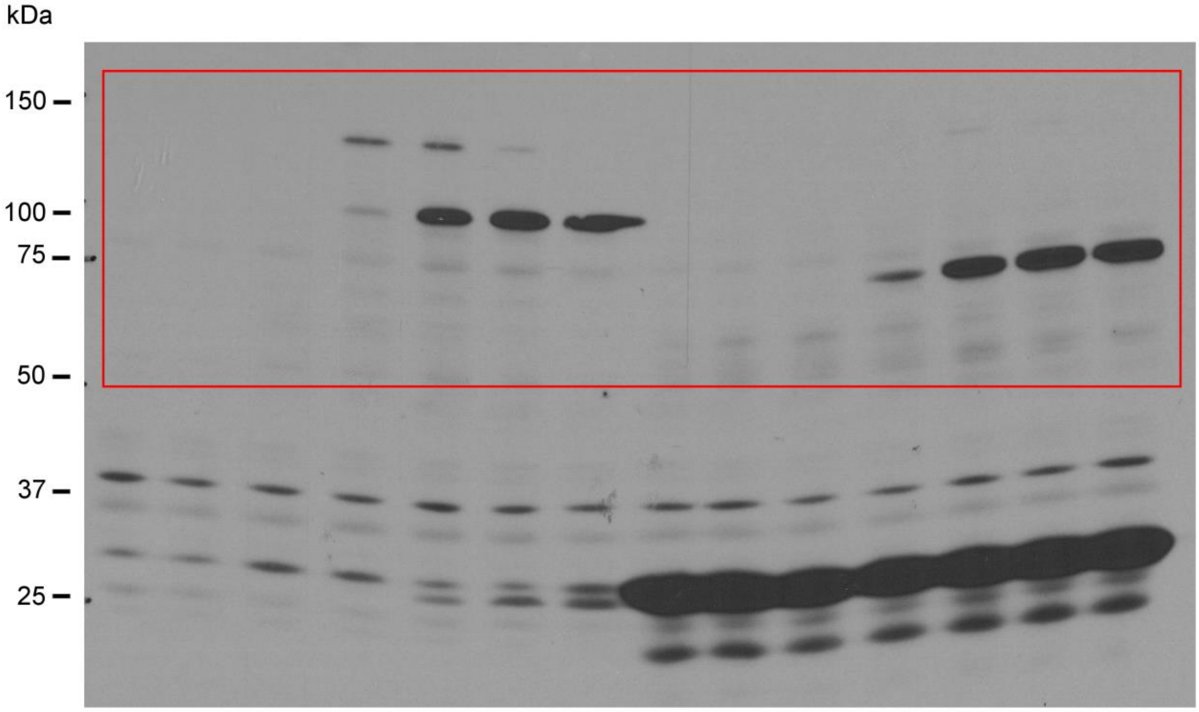


**Supplementary Figure 2g PetΔL4P**

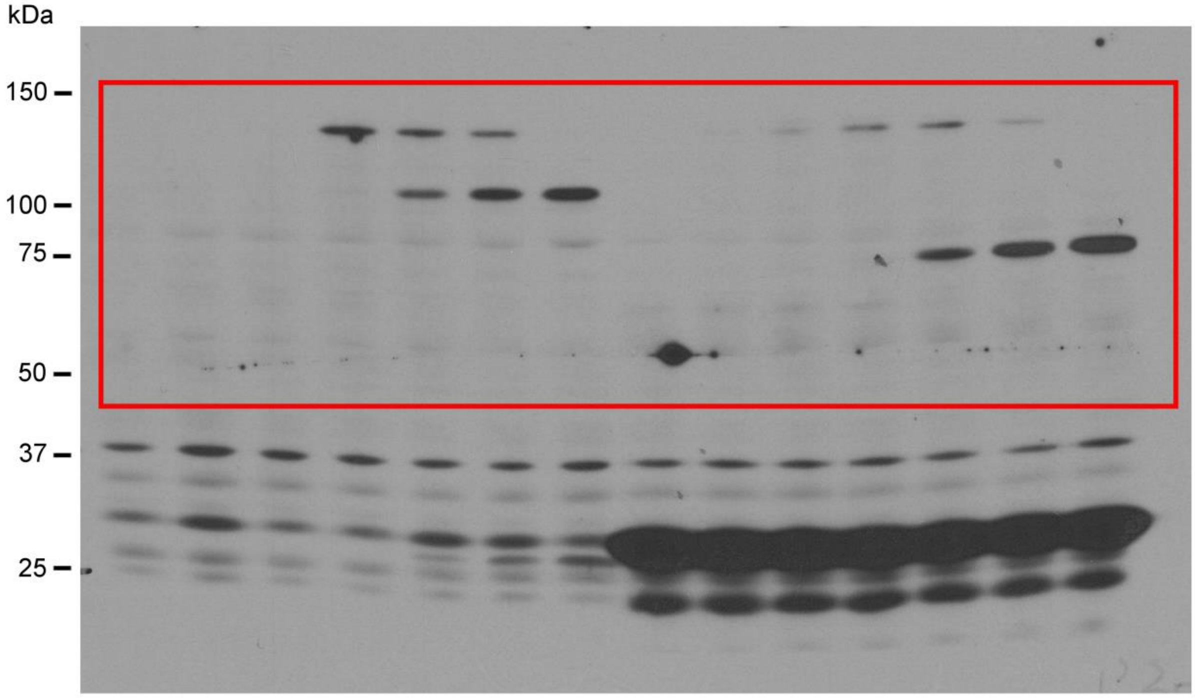

**Supplementary Figure 3 Pet**

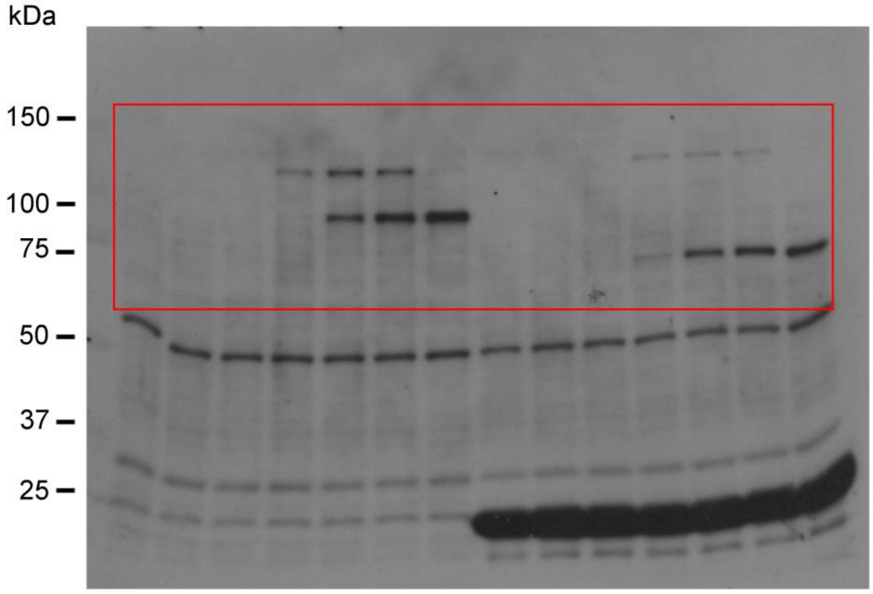


**Supplementary Figure 3 Pet∆L5**

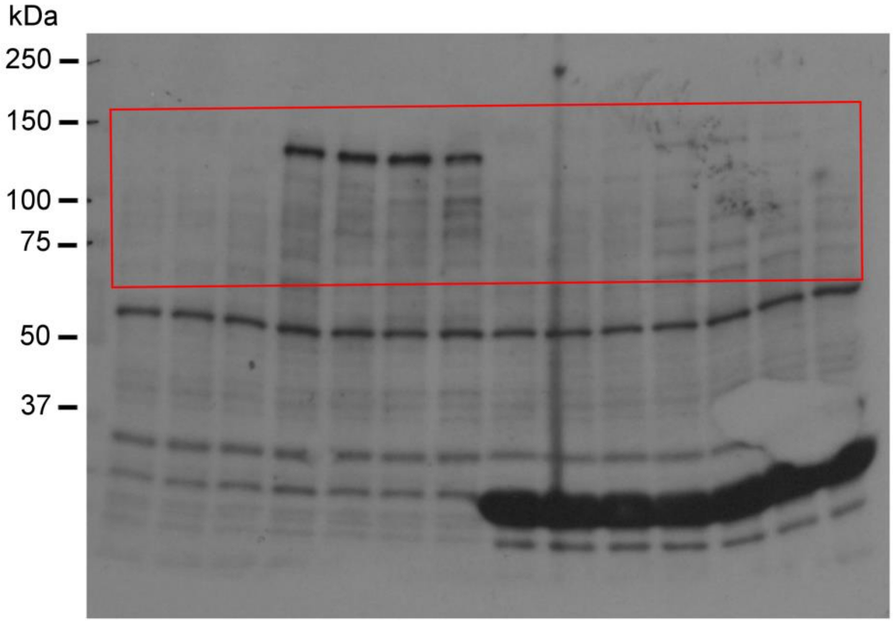

**Supplementary Figure 4b Pet**

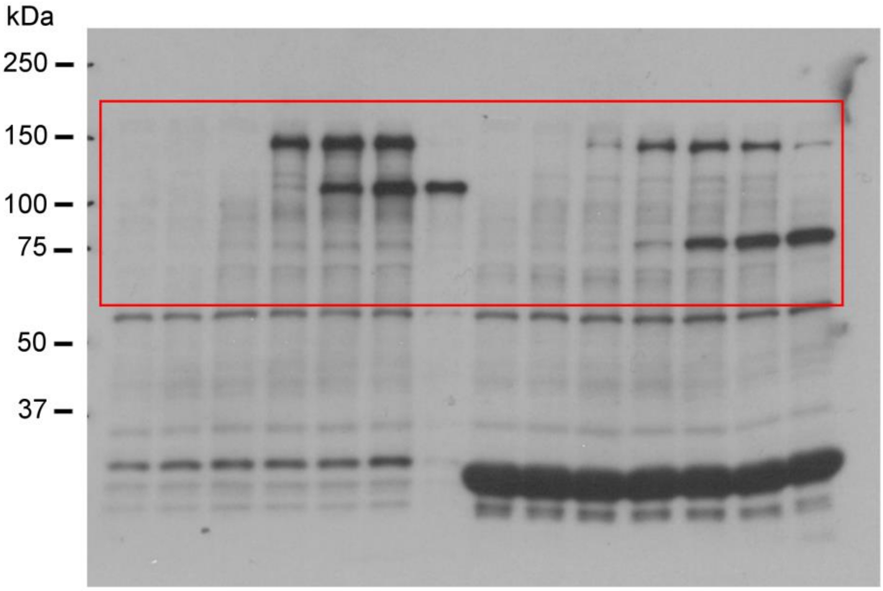


**Supplementary Figure 4b PetL5β1/G**

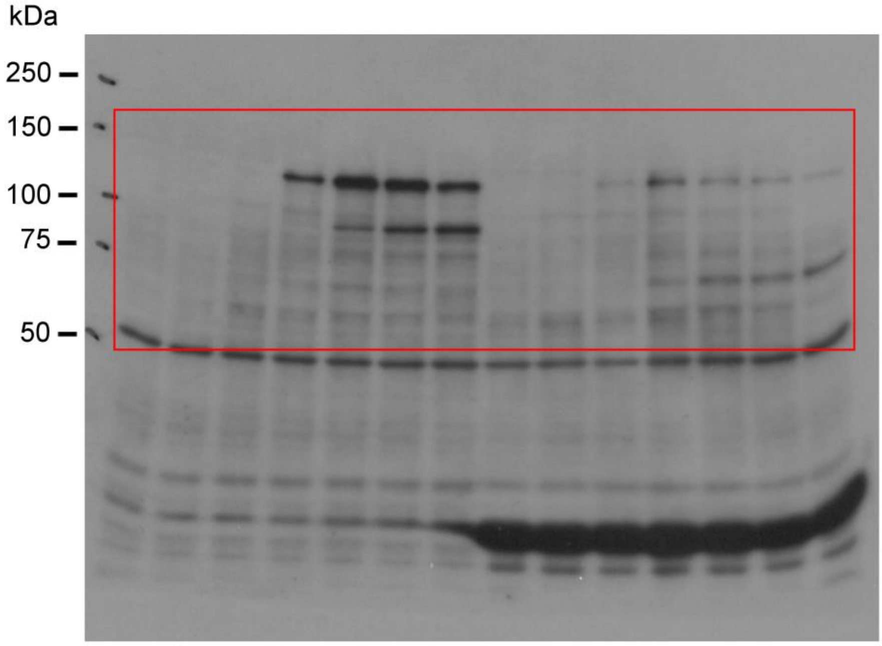

**Supplementary Figure 4b PetL5β1/P**

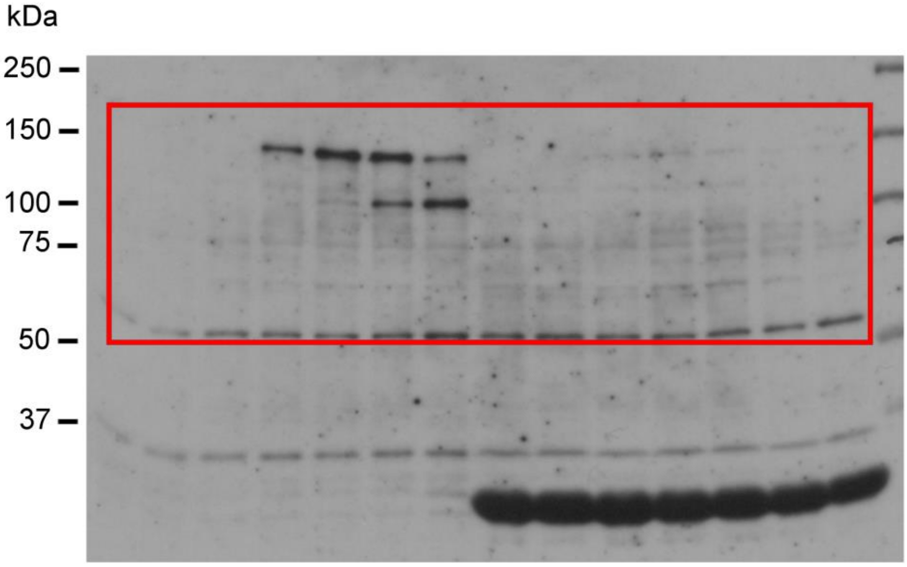


**Supplementary Figure 4b PetL5OmpF**

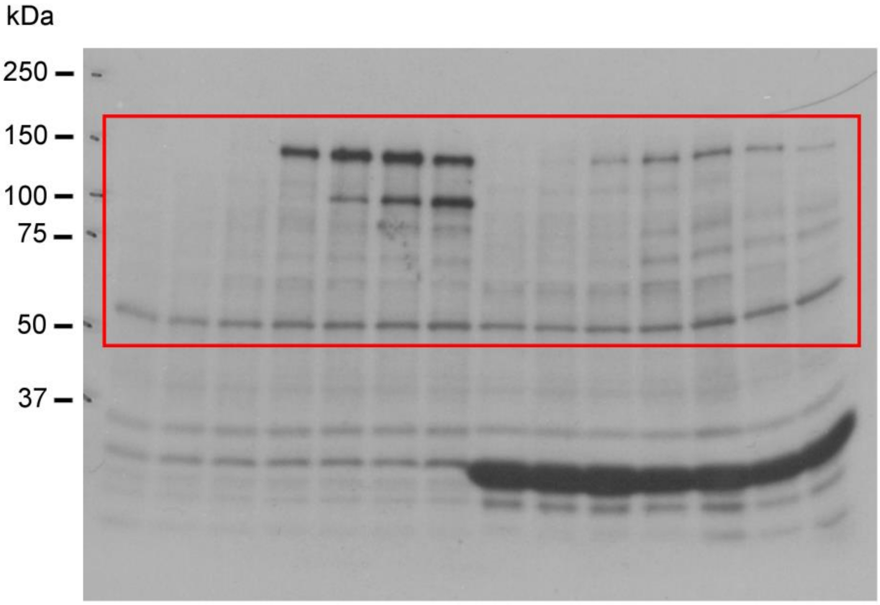

**Supplementary Figure 5b Pet**

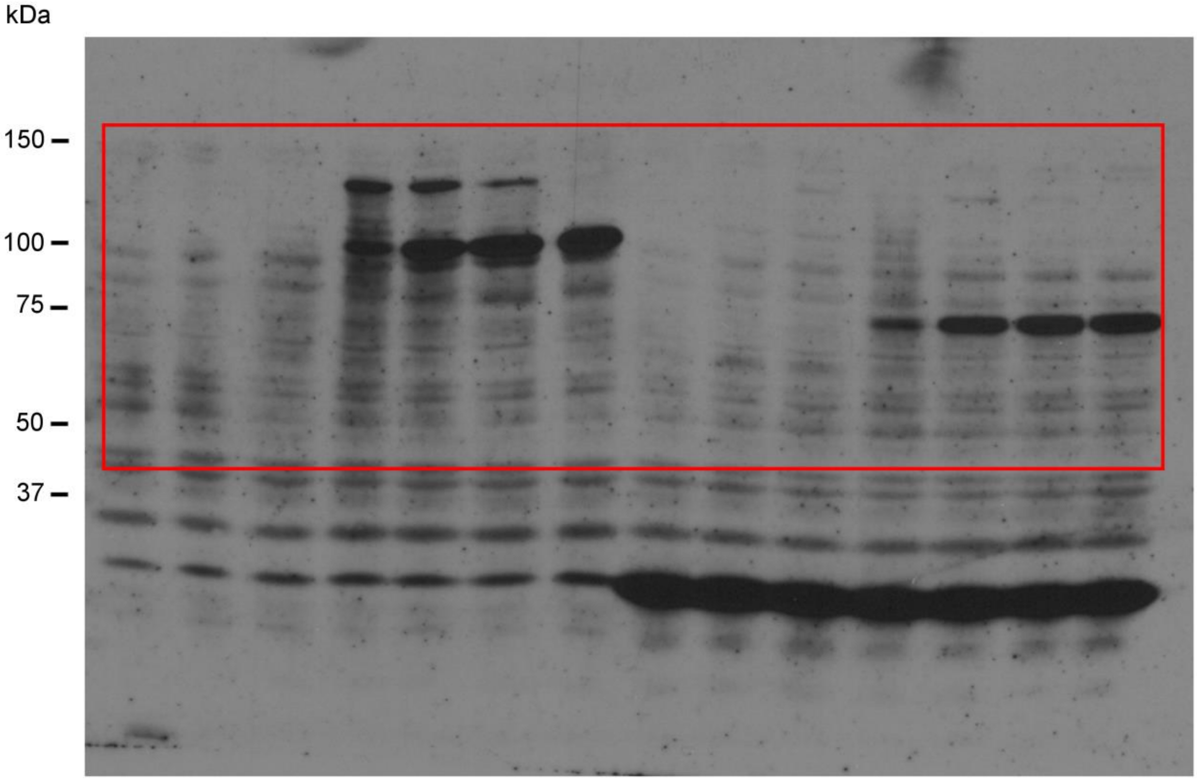


**Supplementary Figure 5b PetL5VLI/N**

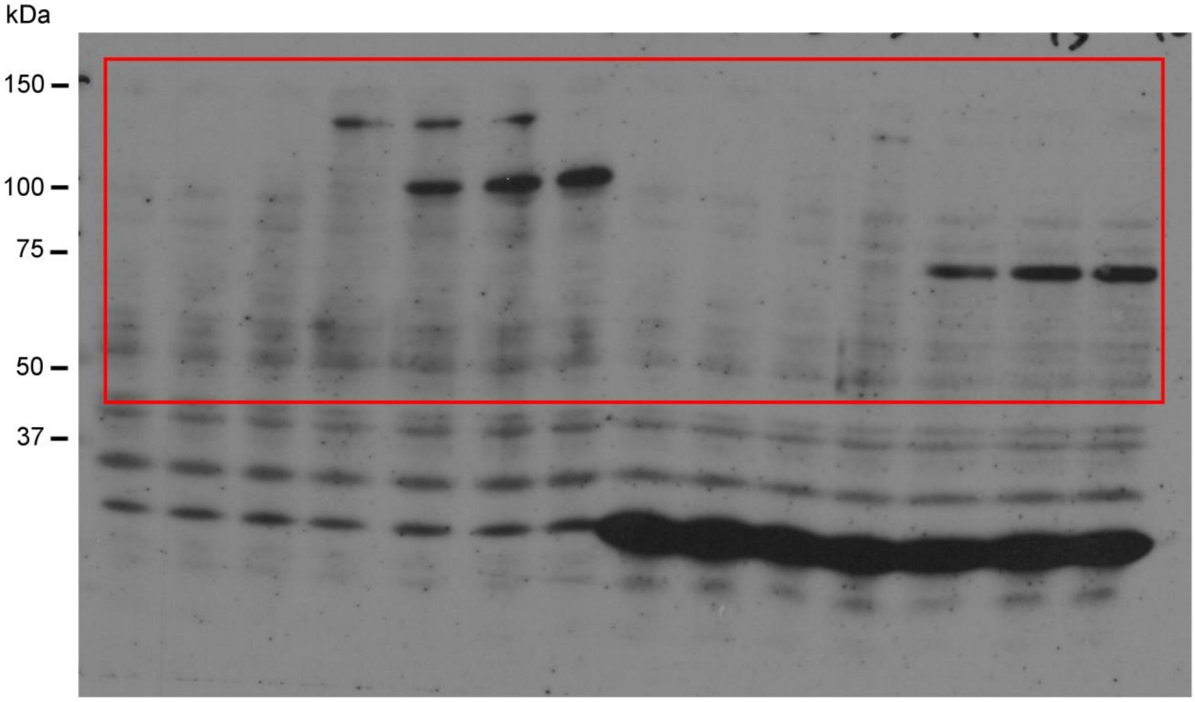

**Supplementary Figure 5b PetL5R/D,E/K**

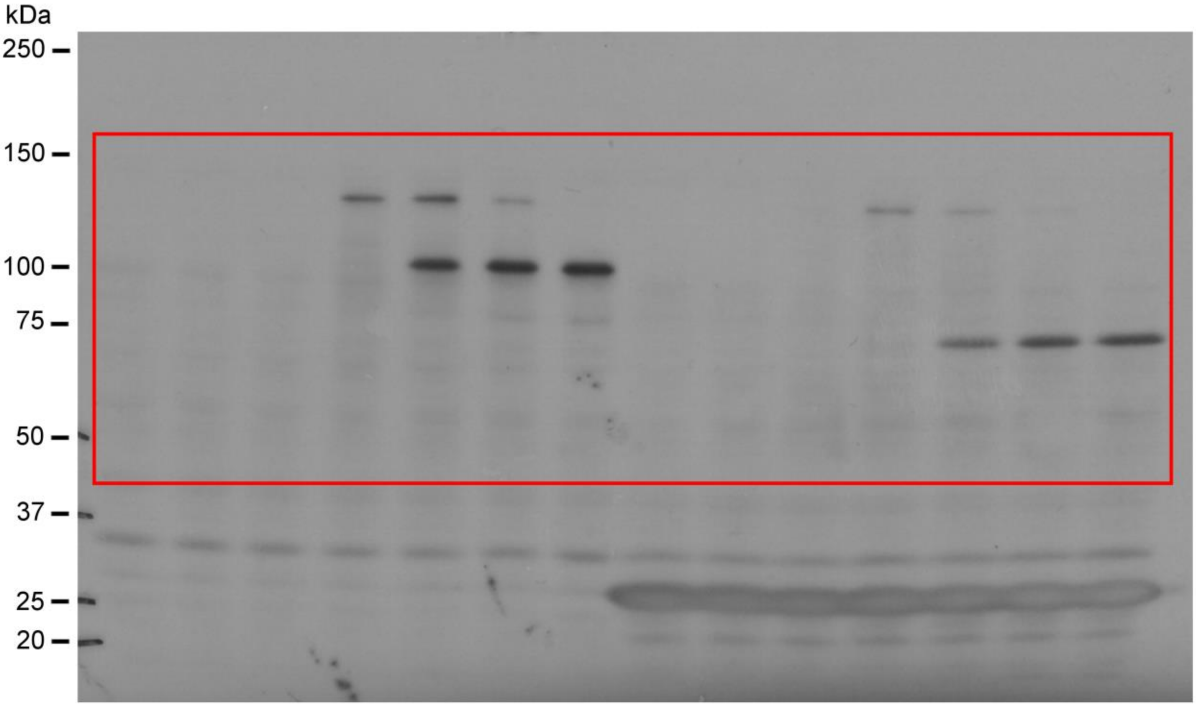


**Supplementary Figure 5b PetL5TD/A**

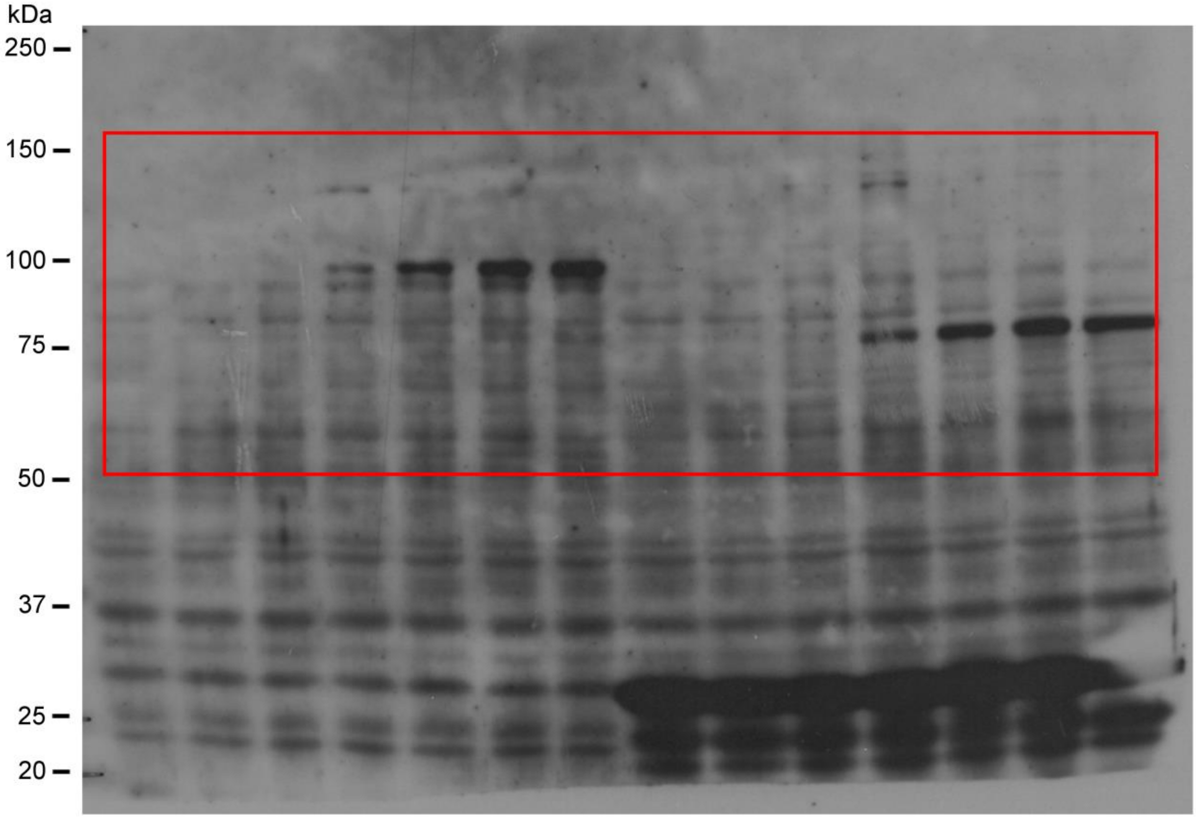

**Supplementary Figure 7d Pet**

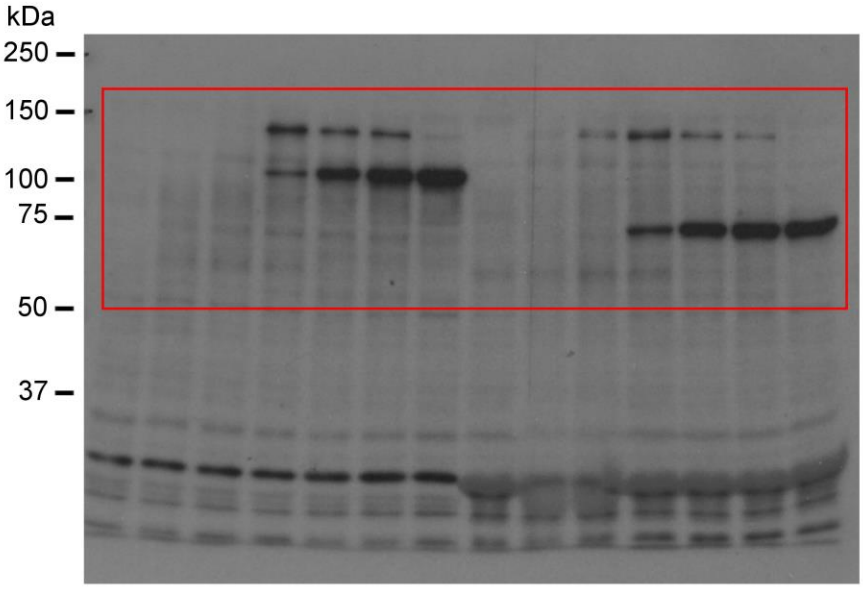


**Supplementary Figure 7d PetL5AIDA-I**

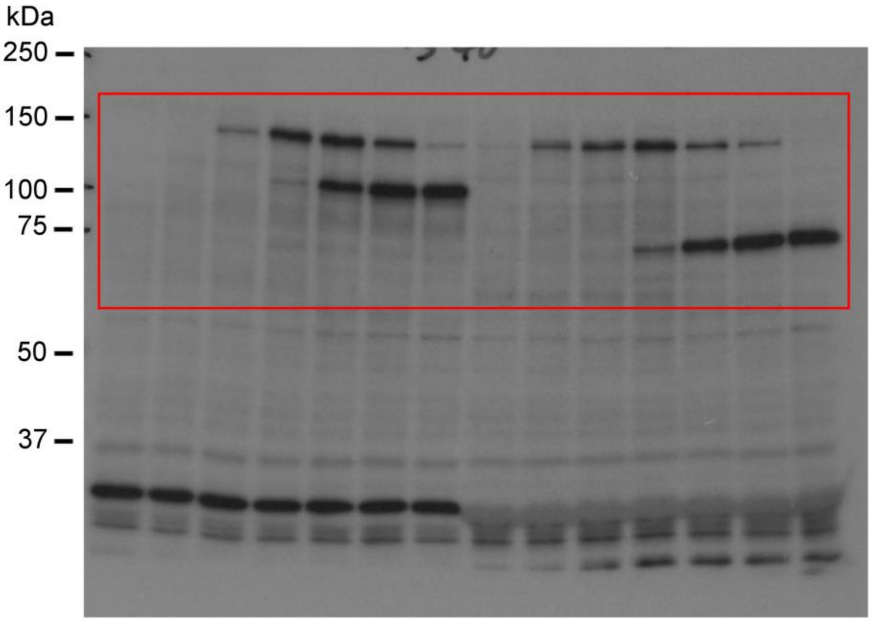

**Supplementary Figure 7d PetL5EstA**

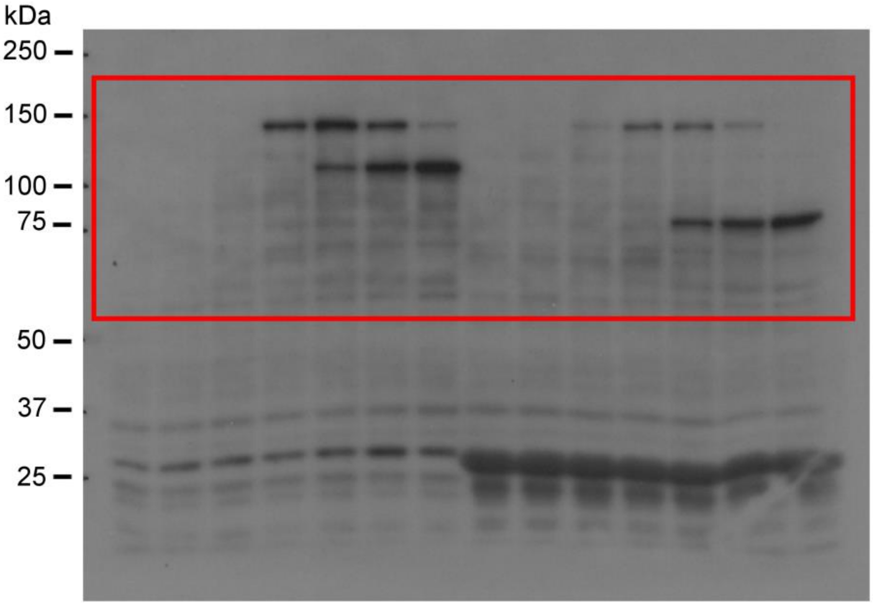


**Supplementary Figure 8a PetΔ1-554 (left)** **PetΔL5Δ1-554 (right)**

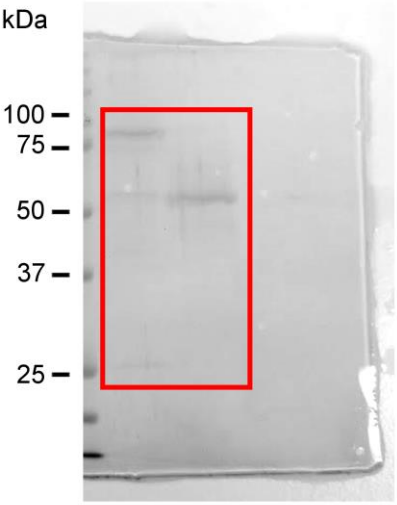


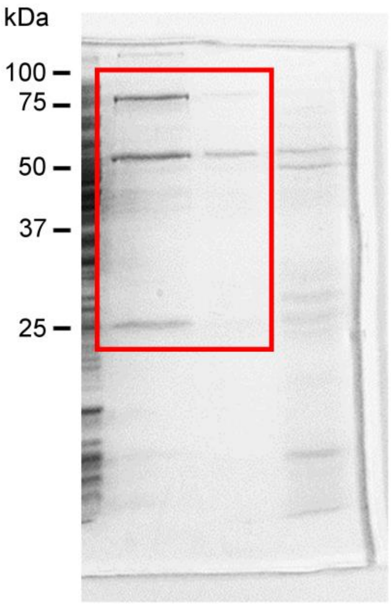

**Supplementary Figure 8b PetΔ1-554 (left)**

**Refold IMAC**

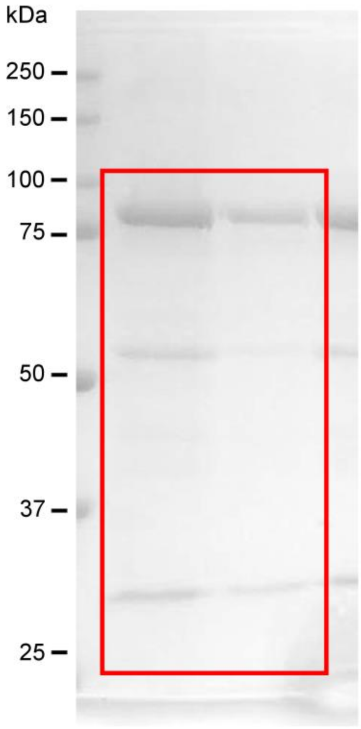


**Purified β-Barrel**

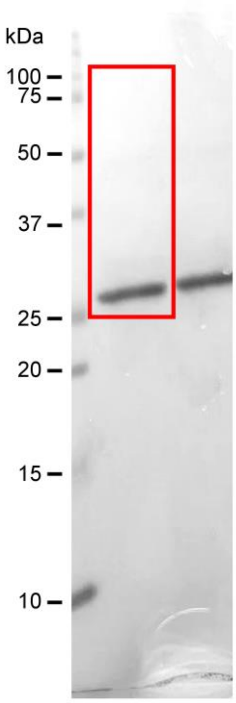


**Supplementary Figure 8b PetΔL5Δ1-554 (right)**

**Refold IMAC**

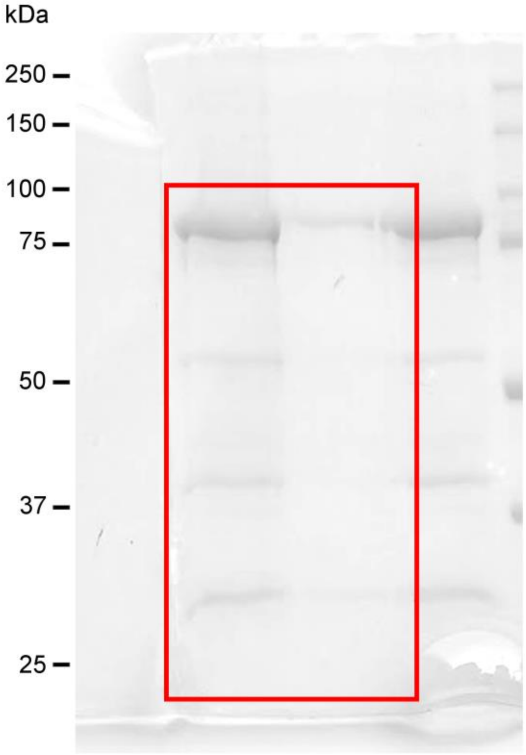


**Purified β-Barrel**

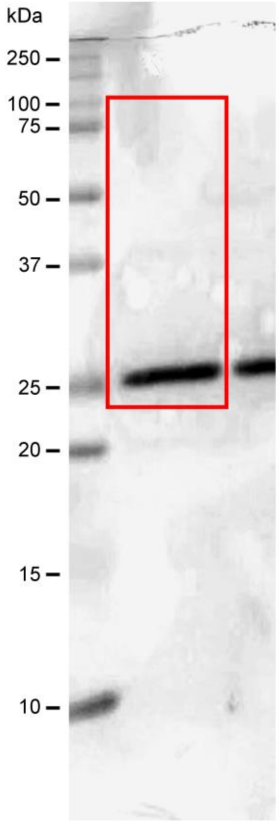

**Supplementary Figure 9 | Raw image files.** The raw image files of the cropped immunoblots and Coomassie-stained gels displayed in the Figures and Supplementary Figures. Sizes (in kDa) are indicated on the left. A red box is used to indicate the portion of the raw image that was cropped and displayed in the indicated Figures and Supplementary Figures.

**Supplementary Table 1. Strains and plasmids used in this study**

| Strain/Plasmid | Relevant description | Reference |
|---|---|---|
| *E. coli* TOP10 | F– *mcr*A Δ(*mrr-hsd*RMS-*mcr*BC) Φ80*lac*ZΔM15 Δ*lac*X74 *rec*A1 *ara*D139 Δ(*ara leu*) 7697 *gal*U *gal*K *rps*L (StrR) *end*A1 *nup*G | Invitrogen |
| *E. coli* BW25113 | *lacI*$^{q}$ *rrnB*$_{T14}$ Δ*lacZ*$_{WJ16}$ *hsdR514* Δ*araBAD*$_{AH33}$ Δ*rhaBAD*$_{LD78}$ | [2] |
| *E. coli* BW25113 *ompT*::*kan* | An in-frame *ompT* knock-out mutant of *E. coli* BW25113 | [2] |
| *E. coli* BL21 (DE3) | F– *omp*T *hsd*SB(rB–, mB–) *gal dcm* (DE3) | Invitrogen |
| pBADHisA | Arabinose-inducible expression vector, ampicillin resistant | Invitrogen |
| pBADPet | pBADHisA derivative expressing *de novo* synthesized Pet | [3] |
| pBADPetΔL3 | pBADPet derivative containing a *de novo* synthesized NgoMIV-AatII fragment that contains three G residues between A$_{1129}$ and T$_{1136}$ to express Pet with a L3 truncation | GenScript / This study |
| pBADPetΔL4 | pBADPet derivative containing a *de novo* synthesized NgoMIV-AatII fragment that contains three G residues between K$_{1176}$ and K$_{1192}$ to express Pet with a full L4 truncation | GenScript / This study |
| pBADPetΔL4P | pBADPet derivative containing a *de novo* synthesized NgoMIV-AatII fragment that contains three G residues between F$_{1178}$ and M$_{1189}$ to express Pet with a partial L4 truncation | GenScript / This study |
| pBADPetΔL5 | pBADPet derivative containing a *de novo* synthesized KpnI-EcoRI fragment that contains three G residues between N$_{1231}$ and E$_{1248}$ to express Pet with a L5 truncation | GenScript / This study |
| pBADPet$^{L5\beta1/G}$ | pBADPet derivative containing a *de novo* synthesized AatII-EcoRI fragment where E$_{1233}$, T$_{1234}$, V$_{1235}$, L$_{1236}$, R$_{1237}$, D$_{1238}$ in β-strand 1 of L5 are mutated to G to assess if these residues play a role in passenger domain folding | GenScript / This study |
| pBADPet$^{L5\beta2/G}$ | pBADPet derivative containing a *de novo* synthesized AatII-EcoRI fragment where E$_{1242}$, K$_{1243}$, R$_{1244}$, I$_{1245}$, K$_{1246}$ in β-strand 2 of L5 are mutated to G to assess if these residues play a role in passenger domain folding | GenScript / This study |
| pBADPet$^{L5Un/G}$ | pBADPet derivative containing a *de novo* synthesized AatII-EcoRI fragment where L$_{1228}$, F$_{1229}$, A$_{1230}$, N$_{1231}$, E$_{1248}$, K$_{1249}$, D$_{1250}$ in the unstructured region beneath β-strands 1 and 2 of L5 are mutated to G to assess if these residues play a role in passenger domain folding | GenScript / This study |
| pBADPet$^{L5VLI/N}$ | pBADPet derivative containing a *de novo* synthesized AatII-EcoRI fragment where V$_{1235}$, L$_{1236}$, I$_{1245}$ in β-strands 1 and 2 of L5 are mutated to N to assess if hydrophobic residues within L5 play a role in passenger domain folding | GenScript / This study |
| pBADPet$^{L5R/D,E/K}$ | pBADPet derivative containing a *de novo* synthesized AatII-EcoRI fragment where R$_{1237}$ and E$_{1233}$ in β-strand 1 of L5 are mutated to D and K, respectively to disturb the putative salt bridges between R$_{1237}$ and E$_{1242,}$ and E$_{1233}$ and R$_{1244}$ | GenScript / This study |

| | | |
|---|---|---|
| pBADPet$^{L5TD/A}$ | pBADPet derivative containing a *de novo* synthesized AatII-EcoRI fragment where $T_{1234}$ and $D_{1238}$ in β-strand 1 of L5 are mutated to A to assess if these residues within L5 play a role in passenger domain folding | GenScript / This study |
| pBADPet$^{L5FadL}$ | pBADPet derivative containing a *de novo* synthesized AatII-EcoRI fragment where β-strands 1 and 2 of L5 are replaced with a loop region from FadL (PDB 3DWO) that forms a β-hairpin structure to assess if a β-hairpin of an unrelated OMP supports passenger domain folding | GenScript / This study |
| pBADPet$^{L5OmpF}$ | pBADPet derivative containing a *de novo* synthesized AatII-EcoRI fragment where β-strands 1 and 2 of L5 are replaced with a loop region from OmpF (PDB 2ZFG) that forms a β-hairpin structure to assess if a β-hairpin of an unrelated OMP supports passenger domain folding | GenScript / This study |
| pBADPet$^{L5\beta1/P}$ | pBADPet derivative where $E_{1233}$, $T_{1234}$, $V_{1235}$, $L_{1236}$, $R_{1237}$, $D_{1238}$ in β-strand 1 of L5 are mutated to P to eliminate potential hydrogen bonds for β-strand augmentation | This study |
| pBADPet$^{L5/5aa}$ | pBADPet derivative containing a *de novo* synthesized AatII-EcoRI fragment where the 6-residue long β-strands were shortened to 5 residues | GenScript / This study |
| pBADPet$^{L5/4aa}$ | pBADPet derivative containing a *de novo* synthesized AatII-EcoRI fragment where the 6-residue long β-strands were shortened to 4 residues | GenScript / This study |
| pBADPet$^{L5/3aa}$ | pBADPet derivative containing a *de novo* synthesized AatII-EcoRI fragment where the 6-residue long β-strands were shortened to 3 residues | GenScript / This study |
| pBADPet$^{L5/2aa}$ | pBADPet derivative containing a *de novo* synthesized AatII-EcoRI fragment where the 6-residue long β-strands were shortened to 2 residues | GenScript / This study |
| pBADPet$^{L5/1aa}$ | pBADPet derivative containing a *de novo* synthesized AatII-EcoRI fragment where the 6-residue long β-strands were shortened to 1 residue | GenScript / This study |
| pBADPet$^{L5EstA}$ | pBADPet derivative containing a *de novo* synthesized AatII-EcoRI fragment where β-strands 1 and 2 of L5 are replaced with a loop region from EstA (PDB 3KVN) that forms a β-hairpin structure to assess if the L5 β-hairpin of a non-SPATE autotransporter supports passenger domain folding | GenScript / This study |
| pBADPet$^{L5AIDA\text{-}I}$ | pBADPet derivative containing a *de novo* synthesized AatII-EcoRI fragment where the L5 β-hairpin is replaced with a loop region from AIDA-I (PDB 4MEE) that forms a β-hairpin structure to assess if the L5 β-hairpin of a non-SPATE autotransporter supports passenger domain folding | GenScript / This study |
| pBADEspP | pBADHisA derivative expressing *de novo* synthesized EspP | GenScript / This study |
| pBADEspPΔL5 | pBADPet derivative containing a *de novo* synthesized SalI-HindIII fragment that contains three G residues between $N_{1236}$ and $E_{1253}$ to express EspP with a L5 truncation | GenScript / This study |
| pET-22b+ | IPTG-inducible expression vector allowing a C-terminal hexahistidine-tag fusion, ampicillin resistant | Novagen |

| | | |
|---|---|---|
| pETPet$^{\Delta 1\text{-}554}$ | Truncated pBADPet derivative containing a hexahistidine-tagged Pet barrel-domain and the last 464 residues of the Pet passenger domain | This study |
| pETPetΔL5$^{\Delta 1\text{-}554}$ | Truncated pBADPetΔL5 derivative containing a hexahistidine-tagged Pet barrel-domain and the last 464 residues of the Pet passenger domain | This study |

Note that the numbers next to the amino acid residues correspond to their position relative to the full-length Pet protein (from $M^1$ to $F^{1295}$) and full-length EspP protein (from $M^1$ to $F^{1300}$).

## Supplementary Table 2. Primers used in this study

| Primer | Sequence | Reference |
|---|---|---|
| NdeIPet464Fw | 5'-GGGAATTC**CATATG**CAGGCGAACTCTATCTCT-3' | This study |
| XhoIPetRv | 5'-CCG**CTCGAG***AGAGCC*GAAAGAGTAACGGAAGTTC-3' | [3] |
| SalIPetFw | 5'-GCTG**GTCGAC**TTCATCGAAAAAAAAGG-3' | This study |
| HindIIIPetRv | 5'-CAGCC**AAGCTT**TTATCAATGATGATGAT-3' | This study |
| MPL5β1Pro | 5'-TTCGACCTGTTCGCTAACGGTCCGCCACCTCCGCC ACCGGCTTCTGGTGAAAAACGTATC-3' | This study |

Restriction enzyme sequences are in bold font, insertion sequences (Gly-Ser linker) are italicized, and site-directed mutations are underlined.

## Supplementary References


1. Edgar, R.C. MUSCLE: multiple sequence alignment with high accuracy and high throughput. *Nucleic Acids Res.* **32**, 1792-1797 (2004).
2. Baba, T. et al. Construction of *Escherichia coli* K-12 in-frame, single-gene knockout mutants: the Keio collection. *Mol Syst Biol* **2**(2006).
3. Leyton, D.L. et al. A mortise–tenon joint in the transmembrane domain modulates autotransporter assembly into bacterial outer membranes. *Nat Commun* **5**(2014).
